# Association between early childhood caries and malnutrition in a sub-urban population in Nigeria

**DOI:** 10.1186/s12887-019-1810-2

**Published:** 2019-11-13

**Authors:** Morenike Oluwatoyin Folayan, Olujide Arije, Maha El Tantawi, Kikelomo Adebanke Kolawole, Mary Obiyan, Olaniyi Arowolo, Elizabeth O. Oziegbe

**Affiliations:** 1 Department of Child Dental Health, Obafemi Awolowo University, Ile-Ife, Nigeria; 2 Institute of Public Health, Obafemi Awolowo University, Ile-Ife, Nigeria; 3 Department of Preventive Dental Sciences, Imam Abdulrahman Bin Faisal University, Dammam, Saudi Arabia; 4 Department of Demography and Statistics, Obafemi Awolowo University, Ile-Ife, Nigeria; 5 Department of Child Dental Health, Obafemi Awolowo University Teaching Hospitals’ Complex, Ile-Ife, Nigeria

**Keywords:** Malnutrition, Early childhood caries, Children, Nigeria

## Abstract

**Background:**

To determine the association between malnutrition and early childhood caries (ECC) in children resident in sub-urban, Nigeria.

**Methods:**

This study was a subset of a larger cross-sectional study the data of which was generated through a household survey conducted in Ile-Ife, Nigeria. The study’s explanatory variable was malnutrition (underweight, overweight, wasting and stunting) and the outcome variable was ECC. Poisson regression analysis was used to determine the association between ECC and malnutrition. Variables (sex, frequency of sugar consumption, maternal knowledge of oral hygiene, oral hygiene status) associated with ECC in the primary study were adjusted for to obtain the adjusted prevalence ratio (APR).

**Results:**

Of the 370 children, 20 (5.41%) were underweight, 20 (5.41%) were overweight, 67 (18.11%) were wasting, 120 (32.43%) were stunted and 18 (4.86%) had ECC. Factors associated with ECC were being stunted, underweight, overweight and fair oral hygiene. The prevalence of ECC was lower in children who were stunted (APR: 0.14; 95% CI: 0.03–0.69; *p* = 0.02), almost seven times higher in children who were overweight (APR: 6.88; 95% CI: 1.83–25.85; *p* < 0.001), and predictively absent in children who were underweight (APR: 0; 95% CI: 0–0; p < 0.001) when compared with children who had normal weight. Non-significant risk indicators for ECC included consuming sugar between meals three times a day or more, having low socioeconomic status and being female.

**Conclusions:**

For this study population, the indicators of malnutrition – being stunted, underweight, overweight - and fair oral hygiene were risk indicators for ECC. The frequency of sugar consumption was not a significant risk indicator when malnutrition was included as an explanatory variable for ECC in the study population.

## Background

Early childhood caries (ECC) – defined as caries in children under 6 years of age who had one or more decayed, missing or filled tooth surfaces in any primary tooth [1] - raises concern because of the possible link with malnutrition (little or too much of some nutrients). ECC leads to pain when not managed, and the pain contributes to poor feeding, nutrient deficiency [2–4] and growth failure (underweight, stunted and wasted) [5, 6]. Also, children with ECC have high levels of pro-inflammatory cytokines [7], which trigger a cascade of inflammatory processes [8] associated with impaired growth and obesity inducing mechanisms [9]. Obesity and stunting also cause changes in the salivary glands and the composition of saliva, which increases the risk for ECC by lowering salivary pH [10].

The association between ECC and malnutrition is however, not clear. Although a few studies have found an association between ECC, body mass index (BMI) [11–13] and growth failure [14–18], others have found no associations [6, 19–21]. Also, while large population-based studies found no association between BMI and ECC [19, 22–24], a longitudinal study indicated that malnutrition causes ECC [25], ECC causes stunting, and underweights have more ECC [26], ECC and obesity are both risk factors for type 2 diabetes [27].

Recent emerging evidence suggest that children with ECC have higher levels of pro-inflammatory cytokines [8]. Cytokines act as mediators of inflammation, infection and immunological processes [28] and they increase with increased severity of ECC [8]. The pro-inflammatory cytokines produces free radicals that generate peroxides, Prostaglandin E2, interleukin 6, tumour necrotizing factor -alpha and cysteinyl leukotrienes – powerful agents in the inflammatory response [29] – which are significantly associated with increased nutritional risk [30]. These inflammatory activities seen in ECC are a component of the pathogenesis of illness-related malnutrition [31, 32] and can impair growth [33]. Severe ECC is associated with iron deficiency anemia [34] which reduces salivary flow [3]; and vitamin D [2, 4], vitamin A, calcium and albumin deficiencies which causes enamel hypoplasia/hypomineralization [35], and lose of the protective effect of iron, vitamins and zinc for the teeth [36].

Malnutrition accounted for 21 to 50% of under-5 deaths and a large proportion of morbidity in low and middle-income countries in 2017 [37]. Malnutrition also contributes to under-5 mortality in Nigeria [38]. The prevalence of malnutrition in Nigeria is higher in rural than in urban areas [39]. For a developing country like Nigeria, identifying factors that contribute to malnutrition may help enhance the design of comprehensive programs to tackle the problem.

The studies on the association between malnutrition and caries in Nigeria were conducted in children older than 5 years [11, 40]. These studies were school-based and found no association between BMI and caries. School-based studies in Nigeria have a problem of representativeness because only 35.6% of children aged 36–59 months receive early childhood education [41], making the samples non-representative of the general population. Consequently, we have conducted a secondary data analysis of a population-based survey of a semi-urban population in Nigeria, to determine if there was any association between ECC and malnutrition in children younger than 6 years. We hypothesized that there is no association between ECC and the nutritional status of children younger than 6 years in Ile-Ife, Nigeria.

## Methods

This study was a sub-study of a primary study that determined the association between digit sucking, caries and periodontitis in 6-months-olds to 12-year-olds resident in Ile-Ife [42]. The primary data were collected through a household survey using a three-level cluster sampling procedure. This involved an initial random selection of enumeration areas within the Ife Central local government area, then the selection of every third household on each street in the randomly selected enumeration areas in the local government area, and finally the selection of an eligible individual from each household. Children who fell within the target age group, were living with their biological parents or legal guardians, and who consented to participate in the study were recruited for the study. Details on the sampling process had been discussed in the primary study [43].

### Ethics consideration

Ethical approval for the study was obtained from the Health Research Ethics Committee of the Obafemi Awolowo University Teaching Hospitals’ Complex Ile-Ife (ERC/2013/07/14). The primary study was conducted according to the guidelines laid down in the Declaration of Helsinki and all procedures involving human subjects/patients were approved by the Institute of Public Health-Health Research Ethics Committee. Written informed consent was obtained from the parents or legal guardian of the study participants.

### Sample size and data collection procedures

The estimated sample size for this secondary data analysis was based on the ECC prevalence of 6.6% generated from the primary study [43]. With a 3% margin of error and a confidence level of 95% precision, a sample size of 263 study participants will be adequate to determine the association between ECC and malnutrition for this secondary data analysis. There were 370 data points extracted from the primary study database for this analysis. This exceeded the needed minimum sample required for this study. We therefore had adequate sample size to determine an association between ECC and malnutrition. The data collection instrument for the main study is included in this manuscript as Additional file 1.

#### Socioeconomic status

Socioeconomic status was assessed because of its role as a determinant of malnutrition. It was measured according to a classification validated for use in Nigeria. The index combines the mother’s education and the father’s occupation to obtain five socio-economic classes for children [44]. Class 1 indicates upper class, class II upper middle class, class III middle class, class IV lower middle class, and class V lower class. For the present analysis, these classes were regrouped into three: high (upper and upper middle classes), middle (middle class) and low (lower middle and lower classes).

#### Mothers’ knowledge of oral health

Folayan et al. [43] described how data on mothers’ knowledge of oral health were collected and handled. The same data were used for this study. Briefly, data were collected using a tool with a possible score ranging from 8 to 40. Scores of 21 and above were categorized as good oral health knowledge, and scores of 20 and below were categorized as poor oral health knowledge.

#### Cariogenic diet

The frequency of consumption of sugary snacks between main meals was collected with a tool described in detail by Folayan et al. [45]. Consumption of sugary snacks between main meals three times a day or more is a significant risk factor for ECC [43].

#### Nutritional status

Nutritional status was assessed based on weight and height of each child according to the International Society for the Advancement of Kinanthropometry standard protocol [46]. Weight was measured in kilograms with an electronic weighing scale and recorded to the nearest tenth of a kilogram. The weighing scale was zero-balanced before the child stepped on it. Each child removed heavy items of clothing and mounted the scale bare footed, standing still and looking straight. Measurements were recorded after fluctuations on the digital screen had stopped

Children’s height was measured in meters with an anthropometer-calibrated customized stadiometer. Each child was asked to step on the standiometer bare footed. Height was the maximum vertical distance from the feet to the vertex of the head, with the head held parallel to the Frankfort plane [47]. In this position, the child looked straight, with arms hanging naturally by the sides and both heels together touching the base of the stadiometer. The heels, buttocks and upper part of the back and back of the head were in contact with the stadiometer. The headpiece was brought down to contact the vertex of the head. The measurement was read to the nearest tenth of a centimeter and converted to a fraction of meter.

Nutritional status was determined according to criteria used by the WHO: height (H), age (A) and weight (W) [47]. Children whose H/A Z-score was below minus two standard deviations from the median of the WHO reference population were considered stunted. Children whose W/H Z-score was below minus two standard deviations from the reference population median were considered wasted. Children whose W/A was below minus two standard deviations from the reference population median were classified as underweight. Children whose W/Z-score was plus one standard deviations from the reference median were considered overweight, and those who were two standard deviations from the reference median were considered obese.

Oral examination for each child was conducted in the homes of the study participants. The participants were examined sitting, under natural light, using sterile dental mirrors and probes by trained dentists who were calibrated on the use of the WHO criteria for caries diagnosis, and the OHI-S index for assessment of the oral hygiene status. Radiographs were not used in this study. The intra-examiner correlation scores ranged between 0.89–0.94, while inter-examiner kappa Cohen scores ranged between 0.82–0.90 for caries detection and OHI-S [42, 43].

#### ECC status

The dmft was identified according to the World Health Organization (WHO) criteria [48]. The dmft score was obtained by adding the d, m and f scores for each child less than 6 years of age. The dmft score was dichotomized into 0 = ECC absent and > 0 = ECC present.

#### Oral hygiene status

Oral hygiene status was assessed with the index of Greene and Vermillion [49]. Good oral hygiene was graded from 0.0 to 1.2; fair oral hygiene from 1.3 to 3.0; and poor oral hygiene from 3.1 and above.

### Data analysis

The mean(SD) age, dmft, oral hygiene score, and proportion of those with ECC, and malnutrition were calculated. Socio-demographic profile of participants in the present study and the original study were compared using chi-square test to establish comparability and ensuring that the present sample is representative of the target population like the original sample.

The association between nutritional status and ECC presence (yes/ no) and confounding variables (age, sex, socio-economic status, frequency of sugar consumption between meals, oral hygiene status and maternal knowledge of oral health) was determined with the chi-square test or Fisher’s exact test where appropriate.

Multivariable Poisson regression models were used to assess the relationship between exposure (nutritional status) and confounders on the outcome variable (presence of ECC measured by prevalence ratio: ECC PR). A modified theoretical model outlined by Folayan et al. [43], was used to assess how various indicators explained ECC PR when they were grouped into blocks. Factors were grouped under three blocks and were successively introduced, one block at a time. Each model included the block of nutritional status (WAZ, HAZ and WHZ) as this was the variable assessed for being a risk indicator for ECC. Other blocks moved into the next model if the *p* value was < 0.2. Model 1 included only the block of nutritional status, while Model 2 included the block of nutritional status and the block of socio-economic and demographic factors (age was excluded because of possible collinearity since it was used to compute nutritional status) since these factors could influence caries and nutritional status. Model 3 included variables that met the inclusion criteria for the next model and they were known moderator variables of the association between ECC and malnutrition namely mother’s knowledge of oral health, frequency of daily consumption of sugar and oral hygiene index. At the introduction of a new block with its factor(s), the variables of all blocks were adjusted simultaneously for each other to produce adjusted prevalence ratios (APRs). To avoid sparse data bias [50] introduced by the low prevalence of ECC, underweight, overweight or their combination, we used robust variance estimation [51].

The cross tabulations were generated with IBM SPSS version 23. The test of association was and the Poisson regression analysis was conducted using Stata 15. The test of significance was set at *P* < 0.05.

## Results

Although 497 children younger than 6 years were recruited into the primary study, only 370 (74.45%) with data on nutritional status were included in this analysis. The mean(SD) age of the 370 children was 44.35 (16.03) months. Also, 20 (5.41%) were underweight, 20 (5.41%) were overweight, 67 (18.11%) were wasting, 120 (32.43%) were stunted and 18 (4.86%) had ECC. The mean (SD) dmft score was 0.14(0.80) and the mean(SD) oral hygiene score was 1.12(1.21).

Table 1 shows the socio-demographic profile of the study participants in the primary study and this study. There was no significant difference in the age (*p* = 0.89), sex (*p* = 0.69), socioeconomic status (*p* = 0.19) and the prevalence of ECC (p = 0.19) of the children in the primary study and the current study.

**Table 1:** Socio-demographic profile of study participants in the primary study and the current study

Variables	Total study participants		X^2^ *p*-value
	Current study *N* = 370 (%)	Primary study *N* = 497 (%)	
Sex			
Male	203 (54.86)	266 (53.52)	0.15 0.69
Female	167 (45.14)	231 (46.48)	
Age Group			
6–23 months	46 (12.44)	72 (14.48)	1.15 0.89
24–35 months	74 (20.00)	95 (19.11)	
36–47 months	88 (23.79)	122 (24.55)	
48–59 months	76 (20.54)	102 (20.53)	
60–71 months	86 (23.33)	106 (21.33)	
Socioeconomic Status			
High	83 (22.43)	129 (25.96)	3.32 0.19
Middle	161 (43.52)	226 (45.47)	
Low	126 (34.05)	142 (28.57)	
Caries			
Present	18 (4.86)	33 (6.64)	1.75 0.19
Absent	352 (95.14)	434 (93.36)	

X^2^represents chi-square

*Factors associated with ECC*: Table 2 shows the factors associated with ECC. ECC was significantly associated with only age (*p* = 0.02): ECC prevalence was highest in the age group 48–59 months.

**Table 2:** Association between ECC and sex, age, socioeconomic status, maternal knowledge of caries prevention, frequency of consumption of sugar, oral hygiene status and malnutrition (*N* = 370)

Variables	ECC status				Test of Association	
	Absent (*N* = 352)		Present (*N* = 18)			
	N	%	N	%		
Sex						
Male	196	55.68	7	38.89	X^2^ *p*-value	1.95 0.16
Female	156	44.32	11	61.11		
Age Group						
6–23 months	46	13.07	0	0.00	X^2^ *p*-value	11.69 0.02*
24–35 months	74	21.02	0	0.00		
36–47 months	83	23.58	5	27.78		
48–59 months	68	19.32	8	44.44		
60–71 months	81	23.01	5	27.78		
Socioeconomic Status						
High	77	21.88	6	33.33	X^2^ *p*-value	3.36 0.19
Middle	157	44.60	4	22.23		
Low	118	33.52	8	44.44		
Mother’s Knowledge of Oral Health						
Poor	109	30.97	6	33.33	X^2^ *p*-value	0.05 0.83
Good	243	69.03	12	66.67		
Frequency of Sugar Consumption in Between Meals						
< 3 times daily	297	84.38	13	72.22	X^2^ *p*-value	1.86 0.17
≥ 3 times daily	55	15.62	5	27.78		
Oral Hygiene Status						
Good	248	70.45	10	65.56	X^2^ *p*-value	2.24 0.29
Fair	96	27.27	8	44.44		
Poor	8	2.28	0	0.00		
Wasting (WAZ)						
Normal	287	81.53	16	88.89	Exact test *p*-value	-0.75
Wasted	65	18.47	2	11.11		
Stunting (HAZ)						
Normal	234	66.48	16	88.89	Exact test *p*-value	-0.07
Stunted	118	33.52	2	11.11		
Underweight/Overweight (WHZ)						
Normal	316	89.78	14	77.78	X^2^ *p*-value	3.14 0.16
Underweight	18	5.11	2	11.11		
Overweight	18	5.11	2	11.11		

*Statistically significant at *P* < 0.05

X^2^represents chi-square

*Risk indicators for ECC*: Table 3 shows the results of the Poisson regression models with robust variance estimation because of sparse data. The significant risk indicators for ECC were stunted, underweight, overweight and fair oral hygiene. None of the study participants had poor oral hygiene. In Model 3 where all confounders were controlled for, the prevalence of ECC was 66% lower in children who were stunted (APR: 0.14; 95% CI: 0.03–0.69; *p* = 0.02) and almost seven times higher in children who were overweight (APR: 6.88; 95% CI: 1.83–25.85; *p* < 0.001) than in children who had normal weight. The APR of ECC in children with underweight was nil (APR: 0; 95% CI: 0–0; p < 0.001) when compared with children who had normal weight. Among the studied children with ECC, none had poor oral hygiene (APR: 0; 95% CI: 0–0; p < 0.001, Tables 2 and 3). Non-significant risk indicators for ECC included consuming sugar between meals three times a day or more (APR: 2.18), having low socioeconomic status (APR: 2.40) and being female (APR: 2.90). This model explained 18% of the differences in the prevalence of ECC observed. The pseudo R2 of the model for ECC increased with the addition of each block, indicating validity of the choice of confounders for caries in the study population.

**Table 3:** Poisson regression model for risk indicators of early childhood caries using robust variance estimation (*N* = 370)

Variables	Model 1 APR (95% CI)	*p*-value	Model 2 APR (95% CI)	*p*-value	Model 3 APR (95% CI)	*p*-value
Wasting (WAZ)						
Normal	1.00	–	1.00	–	1.00	–
Wasted - PR (CI)	0.90 (0.24–3.28)	0.87	0.98 (0.27–3.60)	0.98	1.08 (0.19–6.26)	0.93
Stunting (HAZ)						
Normal	1.00	–	1.00	–	1.00	–
Stunted - PR (CI)	0.26 (0.07–1.05)	0.06	0.25 (0.06–1.04)	0.06	0.14 (0.03–0.69)	0.02*
Underweight/Overweight (WHZ)						
Normal	1.00	–	1.00	–	1.00	–
Underweight- PR (CI)	1.05 (0.17–6.50)	0.96	1.26 (0.20–8.06)	0.81	0.00 (0.00–0.00)	< 0.001*
Overweight- PR (CI)	2.06 (0.51–8.32)	0.31	2.33 (0.55–9.89)	0.25	6.88 (1.83–25.85)	< 0.001*
SEX						
Male			1.00	–	1.00	–
Female- PR (CI)			2.31 (0.89–6.00)	0.09	2.90 (0.91–9.29)	0.07
Socioeconomic status						
High			1.00	–	1.00	–
Middle - PR (CI)			0.39 (0.11–1.39)	0.15	0.41 (0.08–2.02)	0.27
Low - PR (CI)			1.10 (0.37–3.26)	0.86	2.40 (0.62–9.34)	0.21
Oral hygiene status						
Good					1.00	–
Fair- PR (CI)					2.81 (1.02–7.74)	0.05*
Poor - PR (CI)					0.00 (0.00–0.00)	< 0.001*
Frequency of daily consumption of sugar						
< 3 times daily					1.00	–
≥ 3 times daily - PR (CI)					2.18 (0.5–9.53)	0.30
Maternal knowledge of caries prevention						
Poor					1.00	–
Good - PR (CI)					1.24 (0.31–4.93)	0.76
Pseudo R^2^	0.04		0.08		0.18	

*APR* adjusted prevalence ratio, *CI* confidence interval

Model 1 includes four malnutrition factors: wasted, stunted, overweight and underweight. Model 2: includes Model 1 variables plus demographic variables (gender and socioeconomic status). Model 3: includes Model 2 variables plus oral hygiene status, frequency of sugar consumption and maternal knowledge of caries prevention

*Statistically significant at *P* < 0.05

## Discussion

For this study population, our hypothesis was sustained - there was an association between ECC and nutritional status of pre-school children: children who were stunted or underweight had a lower prevalence of ECC and children who were overweight had higher prevalence of ECC. ECC was also associated with oral hygiene status ECC in the study population: children with fair oral hygiene had higher prevalence of ECC than those with good oral hygiene. There were no child with ECC who had poor oral hygiene and so the full extent of the oral hygiene/ECC relationship cannot be assessed. Prior significant factors associated with ECC in the study population – consumption of sugar between meals three times a day or more, being female and having mothers with poor knowledge of oral health – lost their significance in this study, as explanatory factors for ECC.

One of the strengths of the study was that its theoretical model for the study was built on evidence generated from the latest study conducted to identify risk indicators for ECC in Ile-Ife. Also, the study sample is representative of the study population and therefore, the results can be generalized to the study population and to Southwestern Nigeria, a region where the culture and diet – social factors associated with ECC - are similar to that of Ile-Ife [52]. The z score used to determine the nutritional status of the study population is more appropriate for children 2-year-old and younger [53], thereby increasing the validity of the study results [54]. Also, the robust estimation of variance analysis enhanced the validity of the inferential analysis conducted for the subgroups with small sample size.

This study contributes to the sparse literature on the relationship between malnutrition and ECC in sub-Saharan Africa. Although the continent has a high prevalence of malnutrition [55] and a rising caries prevalence [56], the publications on ECC and nutrition are only from Kenya [57, 58], South Africa [59] and Egypt [60]. These previous studies found no significant relationship between ECC and nutritional status. Unlike those studies, we found that being stunted, underweight and overweight were associated with ECC.

The association between ECC and malnutrition had been highlighted in prior studies. Malnutrition results in salivary gland hypofunction, changes in the saliva composition and reduction in its buffering capacity thereby enhancing caries formation [61]. A prior study conducted in Nigeria, had also identified that fair oral hygiene was associated with higher prevalence of ECC [62], while another study highlighted an increase in ECC prevalence as oral hygiene worsened [63]. The calculated estimate for the association between oral hygiene and ECC in this study should be interpreted with caution bearing in mind that there were no children with poor oral hygiene among those with ECC though the overall prevalence of poor oral hygiene was 2.2%. It is important to explore this finding.

We found no significant association between ECC and high sugar consumption in the study population when the analytical model included nutrition status. This may be an indication that the oral hygiene status may be the pathway for caries formation in this population rather than the high frequency of sugar consumption. Malnutrition results in enamel hypoplasia, which creates a nitche environment for plaque retention [61]. Our study finding that malnutrition is associated with fair oral hygiene in the study population further strengthen our hypothesis. The Arantes et al. [64] suggested a more comprehensive assessment of dietary risks related to caries beyond that addressing sugar intake alone. We feel that our study finding – the absence of a relationship between ECC and frequency of sugar consumption in an analysis model that included nutritional status – supports the suggestion of Arantes et al. [64]. We therefore postulate that our observed association between ECC and malnutrition may reflect a complex impact of the household dietary choices – consumption of oil, salt, fat, vegetable, carbohydrate, sugar and micronutrients – on nutritional status and ECC since malnutrition is not limited exclusively to a sugar-caries pathway. Future studies are needed to explore this.

This is a cross-sectional study and therefore liable to the limitations associated with that design such as our inability to establish that malnutrition is a cause of ECC or otherwise. Although we controlled for some confounders by using multivariable analysis techniques [65], we were unable to control for all possible confounders in the absence of suggestive data. An example is that seasonality reportedly affects access to food, with an impact on the prevalence of malnutrition [66]. Also possible confounding variables for ECC such as access to fluoride application, history of breast feeding and feeding at night were not collected in the primary study. The study data may also be limited in its generalizability to the Nigeria as the study was conducted in only one of the 774 local government areas in the country. The proportion of study participants with ECC was low leading to estimates with wide confidence intervals of some associations. Similarly, the low prevalence of poor oral hygiene may be the reason for the observed lower prevalence of ECC association with it. We addressed the issues of low ECC and poor oral hygiene prevalence using robust estimation of variance so that the observed associations are based on sound statistical techniques. Thus, our study findings provide new information on the relationship between ECC and malnutrition in a country where malnutrition is endemic [55]. These findings are important because they produce evidence that is difficult to obtain in other countries where malnutrition prevalence may be lower. The study findings raise a few questions that require further exploration to help improve our understanding of the relationship between ECC and malnutrition.

## Conclusion

We found a significant association between the presence of ECC in children younger than 6 years and being stunted, underweight and overweight in a suburban population with low prevalence of ECC. We also found that there was no association between ECC and the frequency of sugar consumption when the nutritional status of children moderated the relationship. Further studies that examine diet and ECC comprehensively are required. Also, for this study population, further studies are required to help build a stronger predictive model for ECC.

## Supplementary information

**Additional file 1.** Questionnaire for data collection for the primary study. This is the comprehensive study data collection instrument used to generate the data for this study. This study is a subset of a larger primary study that determined the association between caries and oral habits in children 12 years and below.

## Data Availability

All study related data and materials are included in this publication. This study is a sub-study of a larger study the raw data of which is accessible on request.

## Abbreviations

APR: Adjusted Prevalence Ratio
BMI: Body Mass Index
dmft: Decay, missing, filled teeth
ECC: Early Childhood Caries
HAZ: Height for Age Z score
OHI-S: Simplified Oral Hygiene Index
PR: Prevalence Ratio
SD: Standard Deviation
WAZ: Weight for Age Z score
WHO: World Health Organization
WHZ: Weight for Height Z score
