# Supplementary material for: Association between early childhood caries and malnutrition in a sub-urban population in Nigeria
Source: BMC Pediatr. 2019 Nov 13;19:433. doi: 10.1186/s12887-019-1810-2 (PMC6852898; doi:10.1186/s12887-019-1810-2)
Supplement: Supplementary file 1 — Additional file 1. Questionnaire for data collection for the primary study. This is the comprehensive study data collection instrument used to generate the data for this study. This study is a subset of a larger primary study that determined the association between caries and oral habits in children 12 years and below. [file 12887_2019_1810_MOESM1_ESM.doc]

**ASSOCIATION BETWEEN ORAL HABITS AND ORAL HEALTH OF CHILDREN IN IFE CENTRAL LOCAL GOVERNMENT AREA**

# INDIVIDUAL INTERVIEW SCHEDULE FOR CHILDREN AGED 1-12 YEARS AND THEIR LEGAL GUARDIANS

Questionnaire Identification Number |___|___|___|___|___|___|

**Introduction:** My name is ……..…………………..……… I am working for a project being implemented by a team **of** researchers from the Faculty of Dentistry, Obafemi Awolowo University, Ile-Ife. The study is been led by Dr. K.A. Kolawole. We are interviewing children and their parents and care providers in Ife Central LGA in order to find out about certain oral health practices that children engage in, the causes, the consequences of the behaviour on the oral health, and how best to address these behaviours.

**Confidentiality and consent:** I am going to ask you questions some of which may be very personal. Your answers are completely confidential. Your name will not be written on this form. This study involves 1200 children and their parents from Ife Central Local Government Area of Osun State. Your honest answers to these questions will help us better understand what the oral habits in this environment are and how we can work with parents of children who practice these oral habits to stop it. The information collected from you and people like you will help us make adequate plans for clinic and community of children who have oral habits. We would greatly appreciate your help in responding to this survey.

**Interviewer Visit**

|  | **Visit 1** | **Visit 2** | **Visit 3** |
| --- | --- | --- | --- |
| Date |  |  |  |
| Result |  |  |  |
| Interviewer |  |  |  |

**Name______________ Signature________**

**TIME INTERVIEW STARTED_______________**

Name of Coder____________________|___|___| Signature________________ Date______________

SECTION 1: BACKGROUND CHARACTERISTICS OF THE CHILD

| **No.** | **Questions and filters** | **Coding categories** | **Skip to** |
| --- | --- | --- | --- |
| Q101 | **[RECORD SEX OF THE RESPONDENT CHILD]** | Male…………….1  Female………….2 |  |
| Q102 | In what month and year were you born? | Month [___|___]  Don’t know month ………..88  Year [___|___ [___|___]  Don’t know year ………..88 |  |
| Q103 | How old were you as at your last birthday?  **[COMPARE WITH Q102 IF NEEDED AND CORRECT Q103]** | Age in completed years [___|___] |  |
| Q104 | What is your occupation i.e. what kind of work do you mainly do? | Skilled……………...……………....1  Unskilled………………..…………..2  Unemployed…………………...……3  Student………………………..…….4  Others specify[ ]…99 | **→Go to Q106** |
| Q105 | Have you ever attended school? | Yes………………….. ….. 1  No…………………. ….. 2 | **→Go to Q107** |
| Q106 | What is the highest level of school you attended: Quranic only, primary, secondary or higher? | Quranic only………1  Primary …….. …..2  Secondary …….….. 3  Higher ………….. 4 |  |
| Q106A | What is the class/form/year you are currently or completed when you were in school? | Class (Primary) [___]  Form (Secondary) [___]  Year (Tertiary) [___]  Others [___] |  |
| 107 | How long have you been living continuously in this town? | Number of years [___|___]  Record 00 if less than 1 year |  |
| Q108 | What is your religion? | Islam…………. 1  Protestant.……. 2  Catholic……….. 3  Traditional.…….. 4  No religion ……. 5  Others specify[ ]…6  No Response………9 |  |
| Q109 | How old is your mother at her last birthday?  *(please ask directly from parents)* | Age in completed years [___|___] |  |
| Q109a | What is the occupation of your mother i.e. what kind of work do you mainly do? *(please ask directly from parents)* | Skilled (self employed) ..……………....1  Unskilled………………..…………..2  Unemployed…………………...……3  Civil Servant …………………..…….4  Student………………………..…….5  Others specify[ ]…99 |  |
| Q109B | What is the highest level of school your mother attended: *(please ask directly from parents)* | Quranic only………1  Primary …….. …..2  Secondary …….….. 3  Higher ………….. 4 |  |
| Q109C | What is the class/form/year your mother completed when she was in in school?  *(please ask directly from parents)* | Class (Primary) [___]  Form (Secondary) [___]  Year (Tertiary) [___]  Others [___] |  |
| Q110 | How old is your father at her last birthday?*(please ask directly from parents)* | Age in completed years [___|___] |  |
| Q110a | What is the occupation of your father i.e. what kind of work do you mainly do? *(please ask directly from parents)* | Skilled (self employed) ..……………....1  Unskilled………………..…………..2  Unemployed…………………...……3  Civil Servant …………………..…….4  Student………………………..…….5  Others specify[ ]…99 |  |
| Q110B | What is the highest level of school your mother attended: *(please ask directly from parents)* | Quranic only………1  Primary …….. …..2  Secondary …….….. 3  Higher ………….. 4 |  |
| Q110C | What is the class/form/year your mother completed when she was in in school?  *(please ask directly from parents)* | Class (Primary) [___]  Form (Secondary) [___]  Year (Tertiary) [___]  Others [___] |  |
| Q111 | To which ethnic group do you belong? | Birom …………………..1  Bura ……….…………….2  Edo………….…………...3  Efik……….……………. 4  Fulani …….……………..5  Gwari…….……..….……6  Hausa……………....……7  Ibibio…………...…….…8  Idoma………………….….9  Igala………………..…….10  Igbo……………….…….11  Ijaw ……………………..12  Ikwere……………...……13  Itsekiri………….………..14  Kaje…………………….15  Kanuri……………….……16  Okrika…………………….17  Nupe ………………..……18  Shuwa-Arab…………………19  Urhobo……………………20  Tiv………………….……..21  Yoruba…………………….22  Others specify[ ]..23 |  |
| Q112 | Who are you currently living with | Both Parents ……………………1  Mother only………………2  Father only………………3  Mother and stepfather …………4  Father and step mother……………5  Guardian………………….6  Cohabiting ………………….7  Room mates ………………….8  Other [………………] -9 |  |
| Q113 | How many of you are living in your home? | [___|___]  No response ………………….99 |  |
| Q114 | How many children do your parents have? | [___|___]  No response ………………….99 |  |
| Q115 | What is your position among the children your parents have? | [___|___]  No response ………………….99 |  |
| Q116 | Are you one of the children of the person you are living with | Yes..………..…….1  No…………..…...2  No response…………..….99 |  |
| Q117 | How many meals do you eat per day?  **[READ OUT OPTIONS]** [SINGLE CODE ONLY] | Cannot guarantee one meal a day throughout the month………………..1  Only afford one meal a day throughout the month…………………………... 2  Only afford two meals a day throughout the month…………………………….3  Afford three meals a day throughout the month………………………………4  No Response ……………………….5 |  |

**SECTION 2: INFANT FEEDING PRACTICE**

| **No.** | **Questions and filters** | **Coding categories** | **Skip to** |
| --- | --- | --- | --- |
| Q201 | **[ASK MOTHER OF THE CHILD. IF NOT AVAILABLE THEN MOVE TO Q301]**  I would like to ask you about the feeding habits of your child [**USE NAME OF THE CHILD]**. Did you nurse this child from birth? | Yes …………………1  No ………………….2  No Response………..9 | **→Go to Q301** |
| Q201A | How old were you when you gave birth to this child [**USE NAME OF THE CHILD]**? | Years..…… [___]___]  Don’t know…………88  No response………..99 |  |
| Q202 | Did you breast feed this child [**USE NAME OF THE CHILD]**? | Yes………………….1  No…………………..2  No Response………9 | **→Go to Q207** |
| Q202A | Did you time the breast feeding of this child [**USE NAME OF THE CHILD]**? | Yes………………….1  No…………………..2  No Response………9 |  |
| Q203 | For how long did you breastfeed this child [**USE NAME OF THE CHILD]**? | Less than 1 month………….1  Less than 4 months………….2  4 to 6 months………….3  6 to 12 months………….4  12 to 18 months………….5  18 to 24 months………….6  More than 24 month………….7  Cannot remember ………….88  No Response ………….99 |  |
| Q204 | When did you first give water to the child [**USE NAME OF THE CHILD]**?? | At birth ………….1  Within 1 week of birth………….2  Within 1 month of birth………….3  Less than 4 monthsof birth………….4  4 to 6 months of birth………….5  After 6 months of birth………….6  Cannot remember ………….88  No Response ………….99 |  |
| Q205 | When did you first give solid food to the child [**USE NAME OF THE CHILD]**? | At birth ………….1  Within 1 week of birth………….2  Within 1 month of birth………….3  Less than 4 monthsof birth………….4  4 to 6 months of birth………….5  After 6 months of birth………….6  Cannot remember ………….88  No Response ………….99 |  |
| Q206 | Did you breastfeed your child in the night [**USE NAME OF THE CHILD]**? | Yes……………………………...1 No……………………….……2  No Response…………………….9 | **→Go to Q207** |
| Q206a | Do you have often leave the breast in the mouth of the child when you sleep at night? [**USE NAME OF THE CHILD]?** | Yes……………………………...1 No……………………….……2  No Response…………………….9 |  |
| Q206b | What was the reason for your stopping breastfeeding of your child [**USE NAME OF THE CHILD]**? | Had to resume work……………….....1  The breast milk was not enough  for the child………………..……2  I was advice to stop breast feeding  by family………………………...3  I stopped for my health reasons….…...4  Others specify[ ]…….88 No Response……………… …….99 |  |
| Q207 | Did your child use a feeder to feed [**USE NAME OF THE CHILD]**? | Yes……………………………...1 No……………………….……2  No Response…………………….9 | **→Go to Q301** |
| Q207a | Did you time the bottle feeding of this child [**USE NAME OF THE CHILD]**? | Yes……………………………...1 No……………………….……2  No Response…………………….9 |  |
| Q208 | When did your child start to use the feeding bottle [**USE NAME OF THE CHILD]**? | At birth ………….1  Within 1 week of birth………….2  Within 1 month of birth………….3  Less than 4 monthsof birth………….4  4 to 6 months of birth………….5  After 6 months of birth………….6  Cannot remember ………….88  No Response ………….99 |  |
| Q209 | Did you bottle feed your child in the night [**USE NAME OF THE CHILD]**? | Yes……………………………...1 No……………………….……2  No Response…………………….9 |  |
| Q209a | Do you have often leave the bottle in the mouth of the child when you sleep at night?  [**USE NAME OF THE CHILD]?** | Yes……………………………...1 No……………………….……2  No Response…………………….9 |  |

**SECTION 3: ORAL HABITS (1) – DIGIT AND FINGER SUCKING**

| **No.** | **Questions and filters** | **Coding categories** | **Skip to** |
| --- | --- | --- | --- |
| Q301 | From now on, I will ask you specific questions about possible oral habits of your child. Do/Did you child/you ever suck any of the fingers [**USE NAME OF THE CHILD]**?  [**DESCRIBE WHAT DIGIT SUCKING IS TO THE RESPONDENT**] | Yes..……….1  No……… 2  No response ……… 99 | **→Go to Q401** |
| Q302 | Which finger did your child/you suck?  [**USE NAME OF THE CHILD]** | Thumb ………….….1  Digits …………..2  Cannot remember…..…..8 | **→Go to Q303** |
| Q302a | How many fingers did your child/you suck?  [**USE NAME OF THE CHILD]** | 1  2  3  4  Cannot remember…..…..88 |  |
| Q303 | At what age did your child/you start engaging in this habit?  [**USE NAME OF THE CHILD]** | At birth ……………….. 1  Age in months [___|___]  **OR**  Age in years [___|___]  Cannot remember…..…..88 |  |
| Q303a | At what age did your child/you stop engaging in this habit?  [**USE NAME OF THE CHILD]** | Age in years [___|___]  **OR**  Age in months [___|___]  Child is currently still sucking…..…..3  Cannot remember…..…..88 |  |
| Q304 | For how long did your child/you engage in this habit?  [**USE NAME OF THE CHILD]** | Number of years [___|___]  **OR**  Number of months [___|___]  Child is currently still sucking…..…..3  Cannot remember…..…..88 |  |
| Q307 | How often did your child/you engaged in this habit?  [**USE NAME OF THE CHILD]** | Irregularly………….….1  Once a week………….….2  A few (2-3) times a week………….….3  Once a day………….….4  Several times a day………….….5  Cannot remember ………….….88  No response ………….….99 |  |
| Q308 | Each time your child /you suck, how long does it last for?  [**USE NAME OF THE CHILD]** | Less than a minute………….….1  1-5 minutes………….….2  5-10 minutes………….….3  10 – 20 minutes………….….4  20 – 30 minutes………….….5  Almost continually ………….….88  No response ………….….99 |  |
| Q308a | When does your child/you engage with the habit?  **[USE NAME OF THE CHILD]** | Early in the morning………….….1  Before meals ………….….2  When alone………….….3  Before bedtime………….….4  During sleep………….….5  No time pattern observed………….….6  Cannot remember ………….….88  No response ………….….99 |  |
| Q309 | What do you think makes your child/you engage with the habit?  **[USE NAME OF THE CHILD]** | When it is night time……………...….1  When the child wants to breastfeed….2  When the pacifier is not available…….3  When mother is not around………….4  When anxious……………………..….5  When hungry………………..…….….6  Others, please mention……………..88 |  |
| Q310 | When your child/you suck, do you hear the sucking sound? **[USE NAME OF THE CHILD]** | Yes……1  No…..2  Cannot remember ………….….88  No response ………….….99 |  |
| Q311 | When your child stop sucking/you stop sucking, do you hear a popping sound [*please make the sound]*? **[USE NAME OF THE CHILD]** | Yes……1  No…..2  Cannot remember ………….….88  No response ………….….99 |  |
| Q312 | Did you have any concerns about your child/you habit? | Yes……1  No…..2  Cannot remember ………….….88  No response ………….….99 | **→Go to Q313** |
| Q312a | What were you worried about?  [*read out options for the parent and tick all appropriate responses]* | Habit might continue until child  becomes older …..….1  Habit might affect shape of teeth …….2  Habit might affect child appearance….3  Habit might affect child’s in  school performance …..….4  Habit may affect speech of the child….5  Habit may affect feeding of the child ....6  Habit may make people assume  the child is not well brought up….….7  Child’s friends may tease him/her...….9  Others specify[ ]…...88 | **→list (q312b)** |
| Q313 | Did you seek advice from anyone about the habit? | Yes……1  No…..2  Cannot remember ………….….88  No response ………….….99 | **→Go to Q314** |
| Q313a | Who did you seek advice from? | From friends……………...1  From religious leaders…….……….2  From counsellors……………..3  From medical doctor…………….4  From dentists…………….5  Others specify[ ]……...88 |  |
| Q314 | Did you try to stop the habit? | Yes……1  No…..2  Cannot remember ………….….88  No response ………….….99 | **→Go to Q315** |
| Q314a | How did you *(specifically ask parents)* try to stop the habit? | Encouraged peer teasing……………...1  Punishing the child for sucking………2  Application of unpleasant flavoring  substance on the finger….................3  Child broke habit voluntarily....……….4  Interrupting the use of pacifier……......5  Gave rewards for not sucking …....…...6  Wrapping the hand or tape  application to the digit………..…...7  Applying unpleasant flavoring  substance on the pacifier……..….....8  Using a dental appliance……….……...9  Others specify[ ]……...88 | **→Go to after this question Q315** |
| Q314b | How did you *(specifically ask the child if age 8 – 12 years)* try to stop the habit? | Peer teasing…………………...……...1  Punishment from parents……………2  Application of unpleasant flavoring  substance on the finger……….........3  Child broke habit voluntarily…...…….4  Received rewards for not sucking .…...5  Wrapped the hand/finger to  prevent sucking…………….…….6  Using a dental appliance……………...7  Others specify[ ]……...88 |  |
| Q315 | Which effort(s) did you think worked? | Encouraged peer teasing……………...1  Punishing the child for sucking………2  Application of unpleasant flavoring  substance on the finger….................3  Child broke habit voluntarily....……….4  Interrupting the use of pacifier……......5  Gave rewards for not sucking …....…...6  Wrapping the hand or tape  application to the digit………..…...7  Applying unpleasant flavoring  substance on the pacifier……..….....8  Using a dental appliance……….……...9  Others specify[ ]……...88 |  |
| Q315a | Which effort did you think did not worked? | Encouraged peer teasing……………...1  Punishing the child for sucking………2  Application of unpleasant flavoring  substance on the finger….................3  Child broke habit voluntarily....……….4  Interrupting the use of pacifier……......5  Gave rewards for not sucking …....…...6  Wrapping the hand or tape  application to the digit………..…...7  Applying unpleasant flavoring  substance on the pacifier……..….....8  Using a dental appliance……….……...9  Others specify[ ]……...88 |  |
| Q316 | What other habits does the child have? | Hair twisting/pulling……………...1  Sucking a piece of cloth……………...2  Body rocking……………...3  Head rolling……………...4  Head banging……………...5  Nose picking……………...6  Body tics……………...7  Masturbation……………...8  Others specify [ ]..88 |  |

**SECTION 4: ORAL HABITS (2) – TONGUE SUCKING**

| **No.** | **Questions and filters** | **Coding categories** | **Skip to** |
| --- | --- | --- | --- |
| Q401 | Do/Did your child/you suck the tongue [**USE NAME OF THE CHILD]**?  [**DESCRIBE WHAT TONGUE SUCKING IS TO THE RESPONDENT**] | Yes..……….1  No……… 2  No response ……… 99 | **→Go to Q501** |
| Q402 | At what age did your child/you start engaging in this habit?  [**USE NAME OF THE CHILD]** | Age in years [___|___]  **OR**  Age in months [___|___]  Cannot remember…..…..88 |  |
| Q403 | For how long did your child/you engage in this habit?  [**USE NAME OF THE CHILD]** | Number of years [___|___]  **OR**  Number of months [___|___]  Child is still sucking…..…..3  Cannot remember…..…..88 |  |
| Q403A | At what age did your child/you stop engaging in this habit?  [**USE NAME OF THE CHILD]** | Age in years [___|___]  **OR**  Age in months [___|___]  Cannot remember…..…..88 |  |
| Q404 | How often did your child/you engaged in this habit?  [**USE NAME OF THE CHILD]** | Irregularly………….….1  Once a week………….….2  A few (2-3) times a week………….….3  Once a day………….….4  Several times a day………….….5  Cannot remember………….….88  No response………….….99 |  |
| Q405 | Each time your child /you suck, how long does it last for?  [**USE NAME OF THE CHILD]** | Less than a minute………….….1  1-5 minutes………….….2  5-10 minutes………….….3  10 – 20 minutes………….….4  20 – 30 minutes………….….5  Almost continually ………….….88  No response ………….….99 |  |
| Q406 | When does your child/you engage with the habit?  **[USE NAME OF THE CHILD]** | Early in the morning………….….1  Before meals ………….….2  When alone………….….3  Before bedtime………….….4  During sleep………….….5  No time pattern observed ……….….6  Cannot remember ………….….88  No response ………….….99 |  |
| Q407 | What do you think makes your child/you engage with the habit?  **[USE NAME OF THE CHILD]** | When it is night time……………...….1  When the child wants to breastfeed….2  When the pacifier is not available…….3  When mother is not around………….4  When anxious……………………..….5  When hungry…………………..….….6  Others, please mention……………...88 |  |
| Q408 | When your child/you suck, do you hear the sucking sound?  **[USE NAME OF THE CHILD]** | Yes……1  No…..2  Cannot remember ………….….88  No response ………….….99 |  |
| Q409 | Did you have any concerns about your child/you habit? | Yes……1  No…..2  Cannot remember ………….….88  No response ………….….99 | **→Go to Q410** |
| Q409a | What were you worried about? | Habit might continue until child  becomes older …………………….1  Habit might affect shape of teeth …….2  Habit might affect child appearance….3  Habit might affect child’s in  school performance …………..….4  Child’s friends may tease him/her....….5  Others specify[ ]………88 | **→list (q409b)** |
| Q410 | Did you seek advice from anyone about the habit? | Yes……1  No…..2  Cannot remember ………….….88  No response ………….….99 | **→Go to Q411** |
| Q410a | Who did you seek advice from? | From friends……………...1  From religious leaders……….2  From 21counselors……………….3  From medical doctor……………….4  From dentists…………….5  Others specify[ ]…...88 |  |
| Q411 | Did you try to stop the habit? | Yes……1  No…..2  Cannot remember ………….….88  No response ………….….99 | **→Go to Q412** |
| Q411a | How did you *(specifically ask parents)* try to stop the habit? | Encouraged peer teasing……………...1  Punishing the child for sucking………2  Application of unpleasant flavoring  substance on the finger….................3  Child broke habit voluntarily....……….4  Interrupting the use of pacifier……......5  Gave rewards for not sucking …....…...6  Wrapping the hand or tape  application to the digit………..…...7  Applying unpleasant flavoring  substance on the pacifier……..….....8  Using a dental appliance……….……...9  Others specify[ ]……...88 | **→Go to after this question Q412** |
| Q411b | How did you *(specifically ask the child if 8-12 years old)* try to stop the habit? | Peer teasing…………………...……...1  Punishment from parents……………2  Application of unpleasant flavoring  substance on the finger……….........3  Child broke habit voluntarily…...…….4  Received rewards for not sucking .…...5  Wrapped the hand/finger to  prevent sucking…………….…….6  Using a dental appliance……………...7  Others specify[ ]……...88 |  |
| Q412 | Which effort(s) did you think worked? | Encouraged peer teasing……………...1  Punishing the child for sucking………2  Application of unpleasant flavoring  substance on the finger….................3  Child broke habit voluntarily....……….4  Interrupting the use of pacifier……......5  Gave rewards for not sucking …....…...6  Wrapping the hand or tape  application to the digit………..…...7  Applying unpleasant flavoring  substance on the pacifier……..….....8  Using a dental appliance……….……...9  Others specify[ ]……...88 |  |
| Q412a | Which effort did you think did not worked? | Encouraged peer teasing……………...1  Punishing the child for sucking………2  Application of unpleasant flavoring  substance on the finger….................3  Child broke habit voluntarily....……….4  Interrupting the use of pacifier……......5  Gave rewards for not sucking …....…...6  Wrapping the hand or tape  application to the digit………..…...7  Applying unpleasant flavoring  substance on the pacifier……..….....8  Using a dental appliance……….……...9  Others specify[ ]……...88 |  |
| Q413 | What other habits does the child have? | Hair twisting/pulling……………...1  Sucking a piece of cloth……………...2  Body rocking……………...3  Head rolling……………...4  Head banging……………...5  Nose picking……………...6  Body tics……………...7  Masturbation……………...8  Others specify[ ]....88 |  |

**SECTION 5: ORAL HABITS (3) – TONGUE THRUSTING**

| **No.** | **Questions and filters** | **Coding categories** | **Skip to** |
| --- | --- | --- | --- |
| Q501 | Do you child/you thrust your tongue [**USE NAME OF THE CHILD]**?  [**DESCRIBE WHAT TONGUE THRUSTING IS TO THE RESPONDENT**] | Yes..……….1  No……… 2  No response ……… 99 | **→Go to Q601** |
| Q502 | At what age did your child/you start engaging in this habit?  [**USE NAME OF THE CHILD]** | Age in years [___|___]  **OR**  Age in months [___|___]  Cannot remember…..…..88 |  |
| Q503 | For how long did your child/you engage in this habit?  [**USE NAME OF THE CHILD]** | Number of years [___|___]  **OR**  Number of months [___|___]  Child is currently still thrusting…..…..3  Cannot remember…..…..88 |  |
| Q503a | At what age did your child/you stop engaging in this habit?  [**USE NAME OF THE CHILD]** | Age in years [___|___]  **OR**  Age in months [___|___]  Cannot remember…..…..88 |  |
| Q504 | How often did your child/you engaged in this habit?  [**USE NAME OF THE CHILD]** | Irregularly………….….1  Once a week………….….2  A few (2-3) times a week………….….3  Once a day………….….4  Several times a day………….….5  Cannot remember ………….….88  No response ………….….99 |  |
| Q505 | Each time your child /you thrust the tongue, how long does it last for?  [**USE NAME OF THE CHILD]** | Less than a minute………….….1  1-5 minutes………….….2  5-10 minutes………….….3  10 – 20 minutes………….….4  20 – 30 minutes………….….5  Almost continually ………….….88  No response ………….….99 |  |
| Q506 | When does your child/you engage with the habit?  **[USE NAME OF THE CHILD]** | Early in the morning………….….1  Before meals ………….….2  When alone………….….3  Before bedtime………….….4  During sleep………….….5  No time pattern observed ……….….6  Cannot remember ………….….88  No response ………….….99 |  |
| Q507 | What do you think makes your child/you engage with the habit?  **[USE NAME OF THE CHILD]** | When it is night time………….….1  When the child wants to breastfeed….2  When the pacifier is not available.….3  When mother is not around…….….4  When anxious………….….5  When hungry………….….6  Others, please mention………..…..88 |  |
| Q508 | When your child/you thrust, do you hear the thrusting sound?  **[USE NAME OF THE CHILD]** | Yes……1  No…..2  Cannot remember ………….….88  No response ………….….99 |  |
| Q509 | Did you have any concerns about your child/you habit? | Yes……1  No…..2  Cannot remember ………….….88  No response ………….….99 | **→Go to Q510** |
| Q509a | What were you worried about?  [*read out options for the parent and tick all appropriate responses]* | Habit might continue until child  becomes older………………….….1  Habit might affect shape of teeth …….2  Habit might affect child appearance…..3  Habit might affect child’s in  school performance.……………….4  Habit may affect speech of the child…...5  Habit may affect feeding of the child….6  Habit may make people assume the  child is not well brought up…….….7  Child’s friends may tease him/her….....9  Others specify[ ]…….88 | **→list (q509b)** |
| Q510 | Did you seek advice from anyone about the habit? | Yes.……1  No……..2  Cannot remember..….88  No response..….99 | **→Go to Q511** |
| Q510a | Who did you seek advice from? | From friends……………...1  From religious leaders…………….2  From counsellors…………….3  From medical doctor…………….4  From dentists…………….5  Others specify[ ]…….88 |  |
| Q511 | Did you try to stop the habit? | Yes……1  No…..2  Cannot remember ………….….88  No response ………….….99 | **→Go to Q512** |
| Q511a | How did you *(specifically ask parents)* try to stop the habit? | Encouraged peer teasing……………...1  Punishing the child for sucking………2  Application of unpleasant flavoring  substance on the finger….................3  Child broke habit voluntarily....……….4  Interrupting the use of pacifier……......5  Gave rewards for not sucking …....…...6  Wrapping the hand or tape  application to the digit………..…...7  Applying unpleasant flavoring  substance on the pacifier……..….....8  Using a dental appliance……….……...9  Others specify[ ]……...88 | **→Go to after this question Q512** |
| Q511b | How did you *(specifically ask the child if child is 8-12 years old)* try to stop the habit? | Peer teasing………………...….……...1  Punishment from parents…….………2  Application of unpleasant flavoring  substance on the finger……....3  Child broke habit voluntarily…....…….4  Received rewards for not sucking...…...5  Wrapped the hand/finger to  prevent sucking…………………....6  Using a dental appliance……….……...7  Others specify[ ]…..88 |  |
| Q512 | Which effort(s) did you think worked? | Encouraged peer teasing……………...1  Punishing the child for sucking………2  Child broke habit voluntarily……...….3  Interrupting the use of pacifier…...…...4  Gave rewards for not thrusting ….…...5  Using a dental appliance…….………...6  Others specify[ ]……...88 |  |
| Q512a | Which effort did you think did not worked? | Encouraged peer teasing……………...1  Punishing the child for sucking………2  Child broke habit voluntarily……...….3  Interrupting the use of pacifier…...…...4  Gave rewards for not thrusting ….…...5  Using a dental appliance…….………...6  Others specify[ ]……...88 |  |
| Q513 | What other habits does the child have? | Hair twisting/pulling……...1  Sucking a piece of cloth……………...2  Body rocking……………...3  Head rolling……………...4  Head banging……………...5  Nose picking……………...6  Body tics……………...7  Masturbation……………...8  Others specify[ ]…..88 |  |

**SECTION 6: ORAL HABITS (4) – LIP SUCKING**

| **No.** | **Questions and filters** | **Coding categories** | **Skip to** |
| --- | --- | --- | --- |
| Q601 | Do/did you child/you suck your to lip [**USE NAME OF THE CHILD]**?  [**DESCRIBE WHAT LIP SUCKING IS TO THE RESPONDENT**] | Yes..……….1  No……… 2  No response ……… 99 | **→Go to Q701** |
| Q602 | At what age did your child/you start engaging in this habit?  [**USE NAME OF THE CHILD]** | Age in years [___|___]  **OR**  Age in months [___|___]  Cannot remember…..…..88 |  |
| Q603 | For how long did your child/you engage in this habit?  [**USE NAME OF THE CHILD]** | Number of years [___|___]  **OR**  Number of months [___|___]  Child is currently still sucking…..…..3  Cannot remember…..…..88 |  |
| Q603a | At what age did your child/you stop engaging in this habit?  [**USE NAME OF THE CHILD]** | Age in years [___|___]  **OR**  Age in months [___|___]  Cannot remember…..…..88 |  |
| Q604 | How often did your child/you engaged in this habit?  [**USE NAME OF THE CHILD]** | Irregularly………….….1  Once a week………….….2  A few (2-3) times a week………….….3  Once a day………….….4  Several times a day………….….5  Cannot remember ………….….88  No response ………….….99 |  |
| Q605 | Each time your child /you suck, how long does it last for?  [**USE NAME OF THE CHILD]** | Less than a minute………….….1  1-5 minutes………….….2  5-10 minutes………….….3  10 – 20 minutes………….….4  20 – 30 minutes………….….5  Almost continually ………….….88  No response ………….….99 |  |
| Q606 | When does your child/you engage with the habit?  **[USE NAME OF THE CHILD]** | Early in the morning………….….1  Before meals ………….….2  When alone………….….3  Before bedtime………….….4  During sleep………….….5  No time pattern observed ………..….6  Cannot remember ………….….88  No response ………….….99 |  |
| Q607 | What do you think makes your child/you engage with the habit?  **[USE NAME OF THE CHILD]** | When it is night time……………....….1  When the child wants to breastfeed.….2  When the pacifier is not available…….3  When mother is not around…...…..….4  When anxious…………………….….5  When hungry………………….….….6  Others, please mention………….…..88 |  |
| Q608 | When your child/you suck, do you hear the sucking sound?  **[USE NAME OF THE CHILD]** | Yes…...1  No…...2  Cannot remember….88  No response.….99 |  |
| Q609 | Did you have any concerns about your child/you habit? | Yes……1  No..…..2  Cannot remember.….88  No response.….99 | **→Go to Q610** |
| Q609a | What were you worried about?  [*read out options for the parent and tick all appropriate responses]* | Habit might continue until child  becomes older………………….….1  Habit might affect shape of teeth….….2  Habit might affect child appearance.….3  Habit might affect child’s in school  performance…………………….….4  Habit may affect speech of the child.….5  Habit may affect feeding of the child….6  Habit may make people assume the  child is not well brought up……..….7  Child’s friends may tease him/her....….9  Others specify[ ]…...88 | **→list (q609b)** |
| Q610 | Did you seek advice from anyone about the habit? | Yes…..…1  No……..2  Cannot remember...….88  No response...….99 | **→Go to Q611** |
| Q610a | Who did you seek advice from? | From friends..……...1  From religious leaders……….2  From counsellors……….3  From medical doctor……….4  From dentists……….5  Others specify[ ]……...88 |  |
| Q611 | Did you try to stop the habit? | Yes……1  No…..2  Cannot remember ………….….88  No response ………….….99 | **→Go to Q612** |
| Q611a | How did you *(specifically ask parents)* try to stop the habit? | Encouraged peer teasing……………...1  Punishing the child for sucking………2  Application of unpleasant flavoring  substance on the lip…………….…3  Child broke habit voluntarily…...…….4  Interrupting the use of pacifier………5  Gave rewards for not sucking………...6  Using a dental appliance……………...7  Others specify[ ]……...88 | **→Go to after this question Q612** |
| Q611b | How did you *(specifically ask the child if 8-12 years old)* try to stop the habit? | Peer teasing………………...….……...1  Punishment from parents…….………2  Application of unpleasant flavoring  substance on the lip……3  Child broke habit voluntarily……....….4  Received rewards for not sucking...…...5  Using a dental appliance……………...6  Others specify[ ]…...88 |  |
| Q612 | Which effort(s) did you think worked? | Encouraged peer teasing……………..1  Punishing the child for sucking………2  Child broke habit voluntarily……...….3  Interrupting the use of pacifier…….....4  Gave rewards for not sucking………...5  Using a dental appliance……………...6  Others specify[ ]……...88 |  |
| Q612a | Which effort did you think did not worked? | Encouraged peer teasing……………...1  Punishing the child for sucking…….…2  Child broke habit voluntarily……...….3  Interrupting the use of pacifier……......4  Gave rewards for not sucking ………..5  Using a dental appliance……………...6  Others specify[ ]…...88 |  |
| Q613 | What other habits does the child have? | Hair twisting/pulling……………...1  Sucking a piece of cloth……………...2  Body rocking……………...3  Head rolling……………...4  Head banging……………...5  Nose picking……………...6  Body tics……………...7  Masturbation……………...8  Others specify[ ]…..88 |  |

**SECTION 7: ORAL HABITS (5) – LIP BITING**

| **No.** | **Questions and filters** | **Coding categories** | **Skip to** |
| --- | --- | --- | --- |
| Q701 | Do/Did you child/you bite your to lip [**USE NAME OF THE CHILD]**?  [**DESCRIBE WHAT LIP BITING IS TO THE RESPONDENT**] | Yes..……….1  No……… 2  No response ……… 99 | **→Go to Q801** |
| Q702 | At what age did your child/you start engaging in this habit?  [**USE NAME OF THE CHILD]** | Age in years [___|___]  **OR**  Age in months [___|___]  Cannot remember…..…..88 |  |
| Q703 | For how long did your child/you engage in this habit?  [**USE NAME OF THE CHILD]** | Number of years [___|___]  **OR**  Number of months [___|___]  Child is currently still biting…..…..3  Cannot remember…..…..88 |  |
| Q703a | At what age did your child/you stop engaging in this habit?  [**USE NAME OF THE CHILD]** | Age in years [___|___]  **OR**  Age in months [___|___]  Cannot remember…..…..88 |  |
| Q704 | How often did your child/you engaged in this habit?  [**USE NAME OF THE CHILD]** | Irregularly………….….1  Once a week………….….2  A few (2-3) times a week………….….3  Once a day………….….4  Several times a day………….….5  Cannot remember ………….….88  No response ………….….99 |  |
| Q605 | Each time your child /you bite, how long does it last for?  [**USE NAME OF THE CHILD]** | Less than a minute………….….1  1-5 minutes………….….2  5-10 minutes………….….3  10 – 20 minutes………….….4  20 – 30 minutes………….….5  Almost continually ………….….88  No response ………….….99 |  |
| Q706 | When does your child/you engage with the habit?  **[USE NAME OF THE CHILD]** | Early in the morning………….….1  Before meals ………….….2  When alone………….….3  Before bedtime………….….4  During sleep………….….5  No time pattern observed ……….….6  Cannot remember ………….….88  No response ………….….99 |  |
| Q707 | What do you think makes your child/you engage with the habit?  **[USE NAME OF THE CHILD]** | When it is night time…………..…..….1  When the child wants to breastfeed…...2  When the pacifier is not available….….3  When mother is not around……….….4  When hungry……………………....….5  When anxious……………………...….6  Others, please mention………….…..88 |  |
| Q708 | When your child/you bits the lips, does it bleed?  **[USE NAME OF THE CHILD]** | Yes……1  No…..2  Cannot remember ………….….88  No response ………….….99 |  |
| Q709 | Did you have any concerns about your child/you habit? | Yes……1  No…..2  Cannot remember ………….….88  No response ………….….99 | **→Go to Q710** |
| Q709a | What were you worried about?  [*read out options for the parent and tick all appropriate responses]* | Habit might continue until child  becomes older………………..….1  Habit might affect shape of teeth ....….2  Habit might affect child appearance.….3  Habit might affect child’s in  school performance………….….4  Habit may affect speech of the child.….5  Habit may affect feeding of the child….6  Habit may make people assume the  child is not well brought up…...….7  Child’s friends may tease him/her....….9  Others specify[ ]…..88 | **→list (q709b)** |
| Q710 | Did you seek advice from anyone about the habit? | Yes……1  No…..2  Cannot remember ………….….88  No response ………….….99 | **→Go to Q711** |
| Q710a | Who did you seek advice from? | From friends…………………..……...1  From religious leaders………….….….2  From counsellors……………….…….3  From medical doctor……………...….4  From dentists………………………....5  Others specify[ ]…..88 |  |
| Q711 | Did you try to stop the habit? | Yes……1  No…..2  Cannot remember ………….….88  No response ………….….99 | **→Go to Q712** |
| Q711a | How did you *(specifically ask parents)* try to stop the habit? | Encouraged peer teasing……………...1  Punishing the child for sucking…….…2  Application of unpleasant flavoring  substance on the lip….……….3  Child broke habit voluntarily……...….4  Interrupting the use of pacifier…...…...5  Gave rewards for not biting ……..…...6  Using a dental appliance……………...7  Others specify[ ]….88 | **→Go to after this question Q712** |
| Q711b | How did you *(specifically ask the child if can communicate)* try to stop the habit? | Peer teasing…………………....……...1  Punishment from parents…….………2  Application of unpleasant flavoring  substance on the lip…………….....3  Child broke habit voluntarily...……….4  Received rewards for not biting ……...5  Using a dental appliance……………...6  Others specify[ ]…88 |  |
| Q712 | Which effort(s) did you think worked? | Encouraged peer teasing……………...1  Punishing the child for sucking.………2  Child broke habit voluntarily…....…….3  Interrupting the use of pacifier...……...4  Gave rewards for not biting….…….....5  Using a dental appliance…….………...6  Others specify[ ]…88 |  |
| Q712a | Which effort did you think did not worked? | Encouraged peer teasing…....………...1  Punishing the child for sucking………2  Child broke habit voluntarily…...…….3  Interrupting the use of pacifier..……...4  Gave rewards for not biting……...…...5  Using a dental appliance……………...6  Others specify[ ]… 88 |  |
| Q713 | What other habits does the child have? | Hair twisting/pulling……………...1  Sucking a piece of cloth……………...2  Body rocking……………...3  Head rolling……………...4  Head banging……………...5  Nose picking……………...6  Body tics……………...7  Masturbation……………...8  Others specify[ ]…88 |  |

**SECTION 8: ORAL HABITS (6) – NAIL BITING**

| **No.** | **Questions and filters** | **Coding categories** | **Skip to** |
| --- | --- | --- | --- |
| Q801 | Do/Did you child/you bite your to nail [**USE NAME OF THE CHILD]**?  [**DESCRIBE WHAT NAIL BITING IS TO THE RESPONDENT**] | Yes..…..….1  No………2  No response..……99 | **→Go to Q901** |
| Q802 | At what age did your child/you start engaging in this habit?  [**USE NAME OF THE CHILD]** | Age in years [___|___]  **OR**  Age in months [___|___]  Cannot remember…..…..88 |  |
| Q803 | For how long did your child/you engage in this habit?  [**USE NAME OF THE CHILD]** | Number of years [___|___]  **OR**  Number of months [___|___]  Child is currently still biting…..…..3  Cannot remember…..…..88 |  |
| Q803a | At what age did your child/you stop engaging in this habit?  [**USE NAME OF THE CHILD]** | Age in years [___|___]  **OR**  Age in months [___|___]  Cannot remember…..…..88 |  |
| Q804 | How often did your child/you engaged in this habit?  [**USE NAME OF THE CHILD]** | Irregularly………….….1  Once a week………….….2  A few (2-3) times a week………….….3  Once a day………….….4  Several times a day………….….5  Cannot remember ………….….88  No response ………….….99 |  |
| Q805 | Each time your child /you bite your nails, how long does it last for?  [**USE NAME OF THE CHILD]** | Less than a minute………….….1  1-5 minutes………….….2  5-10 minutes………….….3  10 – 20 minutes………….….4  20 – 30 minutes………….….5  Almost continually ………….….88  No response ………….….99 |  |
| Q806 | When does your child/you engage with the habit?  **[USE NAME OF THE CHILD]** | Early in the morning………….….1  Before meals ………….….2  When alone………….….3  Before bedtime………….….4  During sleep………….….5  No time pattern observed …...…….….6  Cannot remember ………….….88  No response ………….….99 |  |
| Q807 | What do you think makes your child/you engage with the habit?  **[USE NAME OF THE CHILD]** | When it is night time………………….1  When the child wants to breastfeed.….2  When the pacifier is not available….….3  When mother is not around……….….4  When hungry………………...…….….5  When anxious………………..…….….6  Others, please mention…….………..88 |  |
| Q808 | When your child/you bits the nails, does the fingers bleed?  **[USE NAME OF THE CHILD]** | Yes……1  No…..2  Cannot remember ….88  No response .….99 |  |
| Q809 | Did you have any concerns about your child/you habit? | Yes……1  No…..2  Cannot remember ………….….88  No response ………….….99 | **→Go to Q810** |
| Q809a | What were you worried about?  [*read out options for the parent and tick all appropriate responses]* | Habit might continue until child  becomes older………………..….1  Habit might affect shape of teeth….….2  Habit might affect child appearance…..3  Habit might affect child’s in school  performance…………………….4  Habit may affect speech of the child.….5  Habit may affect feeding of the child…..6  Habit may make people assume the  child is not well brought up….….7  Child’s friends may tease him/her….....9  Others specify[ ]… 88 | **→list (q809b)** |
| Q810 | Did you seek advice from anyone about the habit? | Yes……1  No..…..2  Cannot remember.….88  No response.….99 | **→Go to Q811** |
| Q810a | Who did you seek advice from? | From friends……………..…………...1  From religious leaders……..………….2  From counsellors………………….….3  From medical doctor……………...….4  From dentists………………………....5  Others specify[ ]…88 |  |
| Q811 | Did you try to stop the habit? | Yes……1  No…..2  Cannot remember ………….….88  No response ………….….99 | **→Go to Q812** |
| Q811a | How did you *(specifically ask parents)* try to stop the habit? | Encouraged peer teasing……………...1  Punishing the child for biting…...……2  Application of unpleasant flavoring  substance on the nail.........................3  Child broke habit voluntarily...……….4  Interrupting the use of pacifier…….....5  Gave rewards for not biting...………...6  Using a dental appliance……………...7  Others specify[ ]…...88 |  |
| Q811b | How did you *(specifically ask the child if child is 8 – 12 years old)* try to stop the habit? | Peer teasing……………....…………...1  Punishment from parents……….……2  Application of unpleasant flavoring  substance on the nail…....................3  Child broke habit voluntarily…...…….4  Received rewards for not biting….…...5  Using a dental appliance……………...6  Others specify[ ]….88 | **→Go to after this question Q812** |
| Q812 | Which effort(s) did you think worked? | Encouraged peer teasing………...…...1  Punishing the child for biting……..…2  Child broke habit voluntarily…..…….3  Interrupting the use of pacifier……....4  Gave rewards for not biting..………...5  Using a dental appliance……………...6  Others specify[ ]…. 88 |  |
| Q812a | Which effort did you think did not work? | Encouraged peer teasing……………...1  Punishing the child for biting…....……2  Child broke habit voluntarily………...3  Interrupting the use of pacifier……….4  Gave rewards for not biting………...5  Using a dental appliance……………...6  Others specify[ ]….88 |  |
| Q813 | What other habits does the child have? | Hair twisting/pulling……………...1  Sucking a piece of cloth……………...2  Body rocking……………...3  Head rolling……………...4  Head banging……………...5  Nose picking……………...6  Body tics……………...7  Masturbation……………...8  Others specify[ ]....88 |  |

**SECTION 9: ORAL HABITS (7) – OBJECT BITING**

| **No.** | **Questions and filters** | **Coding categories** | **Skip to** |
| --- | --- | --- | --- |
| Q901 | Do/Did you child/you bite objects [**USE NAME OF THE CHILD]**?  [**DESCRIBE WHAT OBJECT BITING IS TO THE RESPONDENT**] | Yes..….….1  No………2  No response..……99 | **→Go to Q1001** |
| Q901a | What objects did your child/you bite?  [**USE NAME OF THE CHILD]**? | Pen/pencil..……….1  Clothings ………2  Hair ………3  Needle/toothpick ………4  Others specify[ ]….88 |  |
| Q902 | At what age did your child/you start engaging in this habit?  [**USE NAME OF THE CHILD]** | Age in years [___|___]  **OR**  Number of months [___|___]  Cannot remember…..…..88 |  |
| Q903 | For how long did your child/you engage in this habit?  [**USE NAME OF THE CHILD]** | Age in years [___|___]  **OR**  Age in months [___|___]  Child is currently still biting…..…..3  Cannot remember…..…..88 |  |
| Q903a | At what age did your child/you stop engaging in this habit?  [**USE NAME OF THE CHILD]** | Age in years [___|___]  **OR**  Age in months [___|___]  Cannot remember…..…..88 |  |
| Q904 | How often did your child/you engaged in this habit?  [**USE NAME OF THE CHILD]** | Irregularly………….….1  Once a week………….….2  A few (2-3) times a week………….….3  Once a day………….….4  Several times a day………….….5  Cannot remember ………….….88  No response ………….….99 |  |
| Q905 | Each time your child /you bite your nails, how long does it last for?  [**USE NAME OF THE CHILD]** | Less than a minute………….….1  1-5 minutes………….….2  5-10 minutes………….….3  10 – 20 minutes………….….4  20 – 30 minutes………….….5  Almost continually ………….….88  No response ………….….99 |  |
| Q906 | When does your child/you engage with the habit?  **[USE NAME OF THE CHILD]** | Early in the morning………….….1  Before meals ………….….2  When alone………….….3  Before bedtime………….….4  During sleep………….….5  No time pattern observed ………….6  Cannot remember ………….….88  No response ………….….99 |  |
| Q907 | What do you think makes your child/you engage with the habit?  **[USE NAME OF THE CHILD]** | When it is night time………………….1  When the child wants to breastfeed.….2  When the pacifier is not available….….3  When mother is not around……….….4  When hungry………………...…….….5  When anxious………………..…….….6  Others, please mention…….………..88 |  |
| Q908 | When your child/you bits the nails, does the fingers bleed?  **[USE NAME OF THE CHILD]** | Yes……1  No…..2  Cannot remember ….88  No response .….99 |  |
| Q909 | Did you have any concerns about your child/you habit? | Yes……1  No…..2  Cannot remember ………….….88  No response ………….….99 | **→Go to Q910** |
| Q909a | What were you worried about?  [*read out options for the parent and tick all appropriate responses]* | Habit might continue until child  becomes older………………..….1  Habit might affect shape of teeth….….2  Habit might affect child appearance…..3  Habit might affect child’s in school  performance…………………….4  Habit may affect speech of the child.….5  Habit may affect feeding of the child…..6  Habit may make people assume the  child is not well brought up….….7  Child’s friends may tease him/her….....9  Others specify[ ]… 88 | **→list (q909b)** |
| Q910 | Did you seek advice from anyone about the habit? | Yes……1  No..…..2  Cannot remember.….88  No response.….99 | **→Go to Q911** |
| Q910a | Who did you seek advice from? | From friends……………..…………...1  From religious leaders……..………….2  From counsellors………………….….3  From medical doctor……………...….4  From dentists………………………....5  Others specify[ ]…88 |  |
| Q911 | Did you try to stop the habit? | Yes……1  No…..2  Cannot remember ………….….88  No response ………….….99 | **→Go to Q912** |
| Q911a | How did you *(specifically ask parents)* try to stop the habit? | Encouraged peer teasing……………...1  Punishing the child for biting…...……2  Application of unpleasant flavoring  substance on the nail.........................3  Child broke habit voluntarily...……….4  Interrupting the use of pacifier…….....5  Gave rewards for not biting...………...6  Using a dental appliance……………...7  Others specify[ ]…...88 | **→Go to after this question Q912** |
| Q911b | How did you *(specifically ask the child if can communicate)* try to stop the habit? | Peer teasing……………....…………...1  Punishment from parents……….……2  Application of unpleasant flavoring  substance on the nail…....................3  Child broke habit voluntarily…...…….4  Received rewards for not biting….…...5  Using a dental appliance……………...6  Others specify[ ]….88 |  |
| Q912a | Which effort(s) did you think worked? | Encouraged peer teasing………...…...1  Punishing the child for biting……..…2  Child broke habit voluntarily…..…….3  Interrupting the use of pacifier……....4  Gave rewards for not biting..………...5  Using a dental appliance……………...6  Others specify[ ]…. 88 |  |
| Q912b | Which effort did you think did not work? | Encouraged peer teasing……………...1  Punishing the child for biting…....……2  Child broke habit voluntarily………...3  Interrupting the use of pacifier……….4  Gave rewards for not biting………...5  Using a dental appliance……………...6  Others specify[ ]….88 |  |
| Q913 | What other habits does the child have? | Hair twisting/pulling……………...1  Sucking a piece of cloth……………...2  Body rocking……………...3  Head rolling……………...4  Head banging……………...5  Nose picking……………...6  Body tics……………...7  Masturbation……………...8  Others specify[ ]....88 |  |

**SECTION 10: ORAL HABITS (8) – BRUXISM (TEETH GRINDING)**

| **No.** | **Questions and filters** | **Coding categories** | **Skip to** |
| --- | --- | --- | --- |
| Q1001 | Did you child/you chew your teeth [**USE NAME OF THE CHILD]**?  [**DESCRIBE WHAT BRUXISM IS TO THE RESPONDENT**] | Yes..….….1  No………2  No response..……99 | **→Go to Q1101** |
| Q1002 | At what age did your child/you start engaging in this habit?  [**USE NAME OF THE CHILD]** | Age in years [___|___]  **OR**  Number of months [___|___]  Cannot remember…..…..88 |  |
| Q1003 | For how long did your child/you engage in this habit?  [**USE NAME OF THE CHILD]** | Age in years [___|___]  **OR**  Age in months [___|___]  Child is currently still grinding…..…..3  Cannot remember…..…..88 |  |
| Q1003a | At what age did your child/you stop engaging in this habit?  [**USE NAME OF THE CHILD]** | Age in years [___|___]  **OR**  Age in months [___|___]  Cannot remember…..…..88 |  |
| Q1004 | How often did your child/you engaged in this habit?  [**USE NAME OF THE CHILD]** | Irregularly………….….1  Once a week………….….2  A few (2-3) times a week………….….3  Once a day………….….4  Several times a day………….….5  Cannot remember ………….….88  No response ………….….99 |  |
| Q1005 | Each time your child /you chew the teeth, how long does it last for?  [**USE NAME OF THE CHILD]** | Less than a minute………….….1  1-5 minutes………….….2  5-10 minutes………….….3  10 – 20 minutes………….….4  20 – 30 minutes………….….5  Almost continually ………….….88  No response ………….….99 |  |
| Q1006 | When does your child/you engage with the habit?  **[USE NAME OF THE CHILD]** | Early in the morning………….….1  Before meals ………….….2  When alone………….….3  Before bedtime………….….4  During sleep………….….5  No time pattern observed..……….….6  Cannot remember………….….88  No response………….….99 |  |
| Q1007 | What do you think makes your child/you engage with the habit?  **[USE NAME OF THE CHILD]** | When it is night time………………….1  When the child wants to breastfeed.….2  When the pacifier is not available….….3  When mother is not around……….….4  When hungry………………...…….….5  When anxious………………..…….….6  Others, please mention…….………..88 |  |
| Q1008 | When your child/you grind the teeth, do you hear the sound?  **[USE NAME OF THE CHILD]** | Yes……1  No…..2  Cannot remember ….88  No response .….99 |  |
| Q1009 | Did you have any concerns about your child/you habit? | Yes……1  No…..2  Cannot remember ………….….88  No response ………….….99 | **→Go to Q1010** |
| Q1009a | What were you worried about?  [*read out options for the parent and tick all appropriate responses]* | Habit might continue until child  becomes older………………..….1  Habit might affect shape of teeth….….2  Habit might affect child appearance…..3  Habit might affect child’s in school  performance…………………….4  Habit may affect speech of the child.….5  Habit may affect feeding of the child…..6  Habit may make people assume the  child is not well brought up….….7  Child’s friends may tease him/her….....9  Others specify[ ]… 88 | **→list (q1009b)** |
| Q1010 | Did you seek advice from anyone about the habit? | Yes……1  No..…..2  Cannot remember.….88  No response.….99 | **→Go to Q1011** |
| Q1010a | Who did you seek advice from? | From friends……………..…………...1  From religious leaders……..………….2  From counsellors………………….….3  From medical doctor……………...….4  From dentists………………………....5  Others specify[ ]…88 |  |
| Q1011 | Did you try to stop the habit? | Yes……1  No…..2  Cannot remember ………….….88  No response ………….….99 | **→Go to Q1012** |
| Q1011a | How did you *(specifically ask parents)* try to stop the habit? | Encouraged peer teasing……………...1  Punishing the child for biting…...……2  Application of unpleasant flavoring  substance on the nail.........................3  Child broke habit voluntarily...……….4  Interrupting the use of pacifier…….....5  Gave rewards for not biting...………...6  Using a dental appliance……………...7  Others specify[ ]…...88 | **→Go to after this question Q1012** |
| Q1011b | How did you *(specifically ask the child if child is 8-12 years old)* try to stop the habit? | Peer teasing……………....…………...1  Punishment from parents……….……2  Application of unpleasant flavoring  substance on the nail…....................3  Child broke habit voluntarily…...…….4  Received rewards for not biting….…...5  Using a dental appliance……………...6  Others specify[ ]….88 |  |
| Q1012 | Which effort(s) did you think worked? | Encouraged peer teasing………...…...1  Punishing the child for biting……..…2  Child broke habit voluntarily…..…….3  Interrupting the use of pacifier……....4  Gave rewards for not biting..………...5  Using a dental appliance……………...6  Others specify[ ]…. 88 |  |
| Q1012a | Which effort did you think did not worked? | Encouraged peer teasing……………...1  Punishing the child for biting…....……2  Child broke habit voluntarily………...3  Interrupting the use of pacifier……….4  Gave rewards for not biting………...5  Using a dental appliance……………...6  Others specify[ ]….88 |  |
| Q1013 | What other habits does the child have? | Hair twisting/pulling……………...1  Sucking a piece of cloth……………...2  Body rocking……………...3  Head rolling……………...4  Head banging……………...5  Nose picking……………...6  Body tics……………...7  Masturbation……………...8  Others specify[ ]....88 |  |

**SECTION 11: KNOWLEDGE OF CARIES PREVENTION**

***(To be answered by father)***

| **No.** | **Questions and filters** | **Coding categories** | **Skip to** |
| --- | --- | --- | --- |
| I will be asking you a few questions about what you know on how to care for oral health. All your responses are confidential. I will be depending on your truthful statements to make plans for planning for children in this environment. Please do not guess. Also, do feel free to ask about things you do not understand. Thank you. | | | |
| Q1101 | Fluoridation of drinking water is an effective, safe, and efficient way to prevent holes from forming on the teeth | Strongly agree………….….1  Agree………….….2  Disagree………….….3  Strongly disagree………….….4  Don't know………….….5 |  |
| Q1102 | Use of fluoride containing toothpaste is an effective, safe, and efficient way to prevent holes from forming on the teeth. | Strongly agree………….….1  Agree………….….2  Disagree………….….3  Strongly disagree………….….4  Don't know………….….5 |  |
| Q1103 | The number of times you eat sugar containing food has a great role in producing holes in the teeth | Strongly agree………….….1  Agree………….….2  Disagree………….….3  Strongly disagree………….….4  Don't know………….….5 |  |
| Q1104 | Fissure sealant is effective in the prevention of holes developing in newly erupted molars | Strongly agree………….….1  Agree………….….2  Disagree………….….3  Strongly disagree………….….4  Don't know………….….5 |  |
| Q1105 | Rinsing teeth with a lower amount of water after tooth-brushing reduces the risk of caries | Strongly agree………….….1  Agree………….….2  Disagree………….….3  Strongly disagree………….….4  Don't know………….….5 |  |
| Q1106 | Using fluoride toothpaste is more important than the brushing *per se* for preventing holes from forming on the teeth. | Strongly agree………….….1  Agree………….….2  Disagree………….….3  Strongly disagree………….….4  Don't know………….….5 |  |
| Q1107 | Brushing twice daily with fluoride containing toothpaste is effective for preventing holes from developing in the teeth | Strongly agree………….….1  Agree………….….2  Disagree………….….3  Strongly disagree………….….4  Don't know………….….5 |  |
| Q1108 | It is important to visit the dental clinic regularly as a measure for preventing holes from forming in the teeth. | Strongly agree………….….1  Agree………….….2  Disagree………….….3  Strongly disagree………….….4  Don't know………….….5 |  |

**SECTION 12: ORAL HEALTH BEHAVIOUR**

***(To be answered by Father***)

| **No.** | **Questions and filters** | **Coding categories** | **Skip to** |
| --- | --- | --- | --- |
| Q1401 | How often do you usually brush your teeth? | Irregularly or never………….….1  Once a week………….….2  A few (2-3) times a week………….….3  Once a day………….….4  Twice a day………….….5  More than twice a day………….….6  No response………….….99 |  |
| Q1402 | How often do you use toothpaste containing fluoride when brushing? | Always………….….1  Quiet often………….….2  Seldom………….….3  Not at all………….….4  No response………….….99 |  |
| Q1403 | How often do you floss your teeth? | Irregularly or never………….….1  Once a week………….….2  A few (2-3) times a week………….….3  Once a day………….….4  Twice a day………….….5  More than twice a day………….….6  No response………….….99 |  |
| Q1404 | How often do you eat sugar-containing snacks or drinks between your main meals? | About 3 times a day or more ………….….1  About twice a day ………….….2  About once a day ………….….3  Occasionally; not every day ……….….4  Rarely or never eat between meals .….5  No response………….….99 |  |
| Q1405 | What do you do for your dental check-ups? | I go to a dentist to do that……….….1  I ask my or colleagues to do if for me.2  I do it myself………….….3  There is no need to have check-ups…4  No response………….….99 |  |
| Q1406 | When was your last dental check-up? | Within the last 6 months ………….….1  More than 6 months to one year ago 2  More than 1 to 2 years ago ………3  More than 2 to 5 years ago …………4  More than 5 years ago ………….….5  Never ………….….6  Do not remember ………….….88  No response………….….99 |  |
| Q1407 | **Do you smoke cigarettes?** | No, never ………….….1  No, I used to, but I quit ………….….2  Yes, once a month or less …………3  Yes, a few times (2-3) a month ………4  Yes, a few times (2-3) a week ………5  Yes, once a day or more ………….….6  No response………….….99 |  |

**SECTION 13: KNOWLEDGE OF CARIES PREVENTION**

***(To be answered*** by mother)

| **No.** | **Questions and filters** | **Coding categories** | **Skip to** |
| --- | --- | --- | --- |
| I will be asking you a few questions about what you know on how to care for oral health. All your responses are confidential. I will be depending on your truthful statements to make plans for planning for children in this environment. Please do not guess. Also, do feel free to ask about things you do not understand. Thank you. | | | |
| Q1101 | Fluoridation of drinking water is an effective, safe, and efficient way to prevent holes from forming on the teeth | Strongly agree………….….1  Agree………….….2  Disagree………….….3  Strongly disagree………….….4  Don't know………….….5 |  |
| Q1102 | Use of fluoride containing toothpaste is an effective, safe, and efficient way to prevent holes from forming on the teeth. | Strongly agree………….….1  Agree………….….2  Disagree………….….3  Strongly disagree………….….4  Don't know………….….5 |  |
| Q1103 | The number of times you eat sugar containing food has a great role in producing holes in the teeth | Strongly agree………….….1  Agree………….….2  Disagree………….….3  Strongly disagree………….….4  Don't know………….….5 |  |
| Q1104 | Fissure sealant is effective in the prevention of holes developing in newly erupted molars | Strongly agree………….….1  Agree………….….2  Disagree………….….3  Strongly disagree………….….4  Don't know………….….5 |  |
| Q1105 | Rinsing teeth with a lower amount of water after tooth-brushing reduces the risk of caries | Strongly agree………….….1  Agree………….….2  Disagree………….….3  Strongly disagree………….….4  Don't know………….….5 |  |
| Q1106 | Using fluoride toothpaste is more important than the brushing *per se* for preventing holes from forming on the teeth. | Strongly agree………….….1  Agree………….….2  Disagree………….….3  Strongly disagree………….….4  Don't know………….….5 |  |
| Q1107 | Brushing twice daily with fluoride containing toothpaste is effective for preventing holes from developing in the teeth | Strongly agree………….….1  Agree………….….2  Disagree………….….3  Strongly disagree………….….4  Don't know………….….5 |  |
| Q1108 | It is important to visit the dental clinic regularly as a measure for preventing holes from forming in the teeth. | Strongly agree………….….1  Agree………….….2  Disagree………….….3  Strongly disagree………….….4  Don't know………….….5 |  |

**SECTION 14: ORAL HEALTH BEHAVIOUR**

***(To be answered*** by Mother)

| **No.** | **Questions and filters** | **Coding categories** | **Skip to** |
| --- | --- | --- | --- |
| Q1401 | How often do you usually brush your teeth? | Irregularly or never………….….1  Once a week………….….2  A few (2-3) times a week………….….3  Once a day………….….4  Twice a day………….….5  More than twice a day………….….6  No response………….….99 |  |
| Q1402 | How often do you use toothpaste containing fluoride when brushing? | Always………….….1  Quiet often………….….2  Seldom………….….3  Not at all………….….4  No response………….….99 |  |
| Q1403 | How often do you floss your teeth? | Irregularly or never………….….1  Once a week………….….2  A few (2-3) times a week………….….3  Once a day………….….4  Twice a day………….….5  More than twice a day………….….6  No response………….….99 |  |
| Q1404 | How often do you eat sugar-containing snacks or drinks between your main meals? | About 3 times a day or more ………….….1  About twice a day ………….….2  About once a day ………….….3  Occasionally; not every day ……….….4  Rarely or never eat between meals .….5  No response………….….99 |  |
| Q1405 | What do you do for your dental check-ups? | I go to a dentist to do that……….….1  I ask my or colleagues to do if for me.2  I do it myself………….….3  There is no need to have check-ups…4  No response………….….99 |  |
| Q1406 | When was your last dental check-up? | Within the last 6 months ………….….1  More than 6 months to one year ago 2  More than 1 to 2 years ago ………3  More than 2 to 5 years ago …………4  More than 5 years ago ………….….5  Never ………….….6  Do not remember ………….….88  No response………….….99 |  |
| Q1407 | **Do you smoke cigarettes?** | No, never ………….….1  No, I used to, but I quit ………….….2  Yes, once a month or less …………3  Yes, a few times (2-3) a month ………4  Yes, a few times (2-3) a week ………5  Yes, once a day or more ………….….6  No response………….….99 |  |

**SECTION 15: KNOWLEDGE OF CARIES PREVENTION**

***(To be answered by child age*** 8 to 12 years old)

| **No.** | **Questions and filters** | **Coding categories** | **Skip to** |
| --- | --- | --- | --- |
| I will be asking you a few questions about what you know on how to care for oral health. All your responses are confidential. I will be depending on your truthful statements to make plans for planning for children in this environment. Please do not guess. Also, do feel free to ask about things you do not understand. Thank you. | | | |
| Q1301 | Fluoridation of drinking water is an effective, safe, and efficient way to prevent holes from forming on the teeth | Strongly agree………….….1  Agree………….….2  Disagree………….….3  Strongly disagree………….….4  Don't know………….….5 |  |
| Q1302 | Use of fluoride containing toothpaste is an effective, safe, and efficient way to prevent holes from forming on the teeth. | Strongly agree………….….1  Agree………….….2  Disagree………….….3  Strongly disagree………….….4  Don't know………….….5 |  |
| Q1303 | The number of times you eat sugar containing food has a great role in producing holes in the teeth | Strongly agree………….….1  Agree………….….2  Disagree………….….3  Strongly disagree………….….4  Don't know………….….5 |  |
| Q1304 | Fissure sealant is effective in the prevention of holes developing in newly erupted molars | Strongly agree………….….1  Agree………….….2  Disagree………….….3  Strongly disagree………….….4  Don't know………….….5 |  |
| Q1305 | Rinsing teeth with a lower amount of water after tooth-brushing reduces the risk of caries | Strongly agree………….….1  Agree………….….2  Disagree………….….3  Strongly disagree………….….4  Don't know………….….5 |  |
| Q1306 | Using fluoride toothpaste is more important than the brushing *per se* for preventing holes from forming on the teeth. | Strongly agree………….….1  Agree………….….2  Disagree………….….3  Strongly disagree………….….4  Don't know………….….5 |  |
| Q1307 | Brushing twice daily with fluoride containing toothpaste is effective for preventing holes from developing in the teeth | Strongly agree………….….1  Agree………….….2  Disagree………….….3  Strongly disagree………….….4  Don't know………….….5 |  |
| Q1308 | It is important to visit the dental clinic regularly as a measure for preventing holes from forming in the teeth. | Strongly agree………….….1  Agree………….….2  Disagree………….….3  Strongly disagree………….….4  Don't know………….….5 |  |

**SECTION 16: ORAL HEALTH BEHAVIOUR**

***(To be answered by child age 8 to 12 years or by mother of younger children)***

| **No.** | **Questions and filters** | **Coding categories** | **Skip to** |
| --- | --- | --- | --- |
| Q1601 | How often do you usually brush your teeth? | Irregularly or never………….….1  Once a week………….….2  A few (2-3) times a week………….….3  Once a day………….….4  Twice a day………….….5  More than twice a day………….….6  No response………….….99 |  |
| Q1602 | When do you brush your teeth?  *Read the question and tick all that are applicable. Do not read the options* | Morning before breakfast………….….1  Morning after breakfast………….….2  Afternoon before lunch………….….3  Afternoon after lunch………….….4  Evening before supper………….….5  Evening after supper………….….6  No regular time intervals………….….7  No response………….….99 |  |
| Q1603 | Who brushes your teeth for you? | Myself ………….….1  My mum/guardian brushes for me everyday ……………………………………………2  My mum/guardian brushes for me once a week ………………………………………3  My mum/guardian brushes for me occasionally………………………………..4  Others …………………...…………99 |  |
| Q1604 | What do you use to brush your teeth?  *Read the question and tick all that are applicable. Do not read the options* | Toothbrush ………….….1  Chewing stick………….….2  Salt ………….….3  Cotton wool………….….4  Cloth ………….….5  Charcoal ………….….6  Ground glass………….….7  Others …………………………..…88  No response………….….99 |  |
| Q1605 | How often do you use toothpaste containing fluoride when brushing? | Always………….….1  Quiet often………….….2  Seldom………….….3  Not at all………….….4  No response………….….99 |  |
| Q1606 | How often do you use mouth rinses? | Always………….….1  Quiet often………….….2  Seldom………….….3  Never………….….4  No response………….….99 |  |
| Q1607 | How often do you use topical fluorides? | Always………….….1  Quiet often………….….2  Seldom………….….3  Never………….….4  No response………….….99 |  |
| Q1608 | How often do you floss your teeth? | Irregularly or never………….….1  Once a week………….….2  A few (2-3) times a week………….….3  Once a day………….….4  Twice a day………….….5  More than twice a day………….….6  No response………….….99 |  |
| Q1609 | How often do you eat sugar-containing snacks or drinks between your main meals? | About 3 times a day or more ….….1  About twice a day ….….2  About once a day ….….3  Occasionally; not every day ….….4  Rarely or never eat between meals.….5  No response….….99 |  |
| Q1610 | What do you do for your dental check-ups? | I go to a dentist to do that…….….1  I ask my colleagues to do if for me...2  I do it myself………..….….3  I have access to the school dentist….. .4  There is no need for dental check-up...5  No response………….….99 | **→Go to Q1612** |
| Q1611 | When was your last dental check-up? | Within the last 6 months ………….….1  More than 6 months to one year ago....2  More than 1 to 2 years ago……..….….3  More than 2 to 5 years ago……..….….4  More than 5 years ago …………….….5  Never …………….….6  Do not remember ………….….88  No response………….….99 |  |
| Q1612 | Do you smoke cigarettes? | No, never ………….….1  No, I used to, but I quit ………….….2  Yes, once a month or less ……….….3  Yes, a few times (2-3) a month ….….4  Yes, a few times (2-3) a week …….….5  Yes, once a day or more ………….….6  No response………….….99 |  |

**SECTION 17: MEDICAL AND DIETARY HISTORY OF THE CHILD**

***(To be answered by child age 8 to 12 years or by mother of younger children***)

| **No.** | **Questions and filters** | **Coding categories** | **Skip to** |
| --- | --- | --- | --- |
| We will be asking a few details about the health of the child. Once again, all your responses are confidential. Feel free to ask questions about what you are not certain about. Please respond as truthfully as possible and where you do not have answers, please do let’s know. | | | |
| Q1701 | How often does your child vomit? | Once a month ………………….1  Once in two months ………….….2  Once in three months ………….….3  Once in six months ………….….4  Once in a year ………….….5  Only when ill ………….….6  Don’t know………….….88  No response………….….99 |  |
| Q1701a | How often does your child fall sick | Once a month ………………….1  Once in two months ………….….2  Once in three months ………….….3  Once in six months ………….….4  Once in a year ………….….5  Don’t know………….….88  No response………….….99 |  |
| Q1702 | Does your child vomit after eating food? | Yes ………….….1  No ………….….2  Don’t know………….….88  No response………….….99 |  |
| Q1703 | Does your child eat insufficient or excessive food? | Yes ………….….1  No ………….….2  Don’t know………….….88  No response………….….99 |  |
| Q1704 | Does the child regularly brings back food from the stomach into the mouth? | Yes ………….….1  No ………….….2  Don’t know………….….88  No response………….….99 |  |
| Q1705 | Does the child continue to chew food swallowed after (s)he has finished eating | Yes ………….….1  No ………….….2  Don’t know………….….88  No response………….….99 |  |
| Q1706 | Does the child regularly brings back liquid from the stomach into the mouth? | Yes ………….….1  No ………….….2  Don’t know………….….88  No response………….….99 |  |
| Q1707 | Does the child feel that food is stuck behind the breast bone(chest)? | Yes ………….….1  No ………….….2  Don’t know………….….88  No response………….….99 |  |
| Q1708 | Does the child have burning pains in the chest behind the breast bone? | Yes ………….….1  No ………….….2  Don’t know………….….88  No response………….….99 | **→go to (q1709)** |
| Q1708a | Does the pain increase by bending, stooping, lying down or eating | Yes ………….….1  No ………….….2  Don’t know………….….88  No response………….….99 |  |
| Q1708b | Is the pain more likely or worse in the night? | Yes ………….….1  No ………….….2  Don’t know………….….88  No response………….….99 |  |
| Q1708c | Is the pain reduced by taking medicines like mist mag, gascol, polycol, gelucil? | Yes ………….….1  No ………….….2  Don’t know………….….88  No response………….….99 |  |
| Q1709 | Does the child have pain in the upper part of the stomach? | Yes ………….….1  No ………….….2  Don’t know………….….88  No response………….….99 |  |
| Q1710 | Does the child regularly use medicines for stomach pain such as mist mag, gascol, polycol, gelucil? | Yes ………….….1  No ………….….2  Don’t know………….….88  No response………….….99 |  |
| Q1711 | Does the child take alcohol in any form regularly? | Yes ………….….1  No ………….….2  Don’t know………….….88  No response………….….99 |  |
| Q1712 | Does the eyes and mouth feel dry all the time? | Yes ………….….1  No ………….….2  Don’t know………….….88  No response………….….99 |  |
| Q1713 | Is the child using any drug presently? | Yes ………….….1  No ………….….2  Don’t know………….….88  No response………….….99 | **→go to (q1714)** |
| Q1713a | What drug does the child use regularly? | *Please list* |  |
| Q1714 | Does the child take fruit juices and bottled drinks more than 3 times a week – coke etc? | Yes ………….….1  No ………….….2  Don’t know………….….88  No response………….….99 |  |
| Q1715 | Does the child take any of the following medications more than 3 times a week – Vitamin C, aspirin, Nutri C? | Yes ………….….1  No ………….….2  Don’t know………….….88  No response………….….99 |  |
| Q1716 | Does the child put the food in the mouth for a long time before swallowing? | Yes ………….….1  No ………….….2  Don’t know………….….88  No response………….….99 |  |
| Q1717 | Does the child spend lots of time around a battery charger? | Yes ………….….1  No ………….….2  Don’t know………….….88  No response………….….99 |  |
| Q1718 | Does your child complain of pain from the mouth? | Yes ………….….1  No ………….….2  Don’t know………….….88  No response………….….99 | **→go to (q1719)** |
| Q1718a | Does the child complain of pain when eating? | Yes ………….….1  No ………….….2  Don’t know………….….88  No response………….….99 |  |
| Q1718b | Does the child complain of pain when drinking hot or cold water? | Yes ………….….1  No ………….….2  Don’t know………….….88  No response………….….99 |  |
| Q1718c | Does the child complain of pain that makes it difficult for him/her to sleep? | Yes ………….….1  No ………….….2  Don’t know………….….88  No response………….….99 |  |
| Q1719 | Has the ever had radiotherapy of the head and neck? | Yes ………….….1  No ………….….2  Don’t know………….….88  No response………….….99 |  |

**Q1720**

**DRAW A DIETARY CHART FOR 2 DAYS RECALL AND ONE PRESENT DAY**

| Mealtimes | Day 1 | Day 2 | Day 3 |
| --- | --- | --- | --- |
| Before breakfast |  |  |  |
| Breakfast |  |  |  |
| In between meals |  |  |  |
| Lunch |  |  |  |
| In between meals |  |  |  |
| Supper |  |  |  |
| After supper |  |  |  |

**Result of dietary analysis**

________________________________________________________

**SECTION 18**

**CFSS-DS**

*Ask mothers to estimate the level of fear the child (for 6-7 years old only) would perceive in the situations described below. (If child is 8-12 years, ask the child the questions directly)*

|  |  | **Not afraid at all** | **A little afraid** | **A fair amount** | **Pretty much afraid** | **Very afraid** |
| --- | --- | --- | --- | --- | --- | --- |
| 1801 | Dentists |  |  |  |  |  |
| 1802 | Doctors |  |  |  |  |  |
| 1803 | Injections |  |  |  |  |  |
| 1804 | Having someone examine their mouth |  |  |  |  |  |
| 1805 | Having someone open their mouth |  |  |  |  |  |
| 1806 | Having a stranger touch them |  |  |  |  |  |
| 1807 | Having someone examine them |  |  |  |  |  |
| 1808 | The dentist drill |  |  |  |  |  |
| 1809 | The sight of the dentists drilling |  |  |  |  |  |
| 1810 | The noise of the dentist drilling |  |  |  |  |  |
| 1811 | Having someone put instruments in his/her mouth |  |  |  |  |  |
| 1812 | Choking |  |  |  |  |  |
| 1813 | Having to go to the hospital |  |  |  |  |  |
| 1814 | People in white uniforms |  |  |  |  |  |
| 1815 | Having the nurse clean their mouth |  |  |  |  |  |

**SECTION 19**

**REVISED CHILD MANIFEST ANXIETY SCALE**

*Ask mothers to estimate the level of fear the child (for 6-7 years old only) would perceive in the situations described below. (If child is 8-12 years, ask the child the questions directly)*

|  |  | **Yes** | **No** |
| --- | --- | --- | --- |
| 1901 | You have trouble making up your mind |  |  |
| 1902 | You get nervous when things do not go the right way for you |  |  |
| 1903 | Others seem to do things easier than you can |  |  |
| 1904 | I like everyone you know |  |  |
| 1905 | Often you have trouble getting your breath |  |  |
| 1906 | You worry a lot of the time |  |  |
| 1907 | You am afraid of a lot of things |  |  |
| 1908 | You am always kind |  |  |
| 1909 | You get very upset easily |  |  |
| 1910 | You worry about what your parents will say to you |  |  |
| 1911 | You feel that others do not like the way you do things |  |  |
| 1912 | You always have good manners |  |  |
| 1913 | It is hard for you to get to sleep at night |  |  |
| 1914 | You worry about what other people think about you |  |  |
| 1915 | You feel alone even when there are people with you |  |  |
| 1916 | You am always good |  |  |
| 1917 | Often you feel sick in the stomach |  |  |
| 1918 | Your feelings get hurt easily |  |  |
| 1919 | Your hands feel sweaty |  |  |
| 1920 | You am always nice to everyone |  |  |
| 1921 | You am tired a lot |  |  |
| 1922 | Yu worry about what is going to happen |  |  |
| 1923 | Other children are happier than you are |  |  |
| 1924 | You tell the truth every single time |  |  |
| 1925 | You have bad dreams |  |  |
| 1926 | Your feelings get hurt easily when you am fussed at |  |  |
| 1927 | You feel someone will tell you you do things the wrong way |  |  |
| 1928 | You never get angry |  |  |
| 1929 | You wake up scared some of the time |  |  |
| 1930 | You worry when you go to bed at night |  |  |
| 1931 | It is hard for you to keep your mind on your schoolwork |  |  |
| 1932 | You never say things that you shouldn’t |  |  |
| 1933 | You wriggle in your seat a lot |  |  |
| 1934 | Yo am nervous |  |  |
| 1935 | A lot of people are against you |  |  |
| 1936 | You never lie |  |  |
| 1937 | Yu often worry about something bad happening to you |  |  |

**SECTION 20**

**CFSS**

Ask mothers to estimate the level of fear the child (*for 6-7 years old only*) would perceive in the situations described below. (*If child is 8-12 years, ask the child the questions directly)*

|  |  | **Not afraid at all** | **A little afraid** | **A fair amount** | **Pretty much afraid** | **Very afraid** |
| --- | --- | --- | --- | --- | --- | --- |
| 2001 | Having teeth out |  |  |  |  |  |
| 2002 | Injections |  |  |  |  |  |
| 2008 | The dentist drill |  |  |  |  |  |
| 2009 | Meeting the dentist |  |  |  |  |  |
| 2011 | Having someone look into his/her mouth |  |  |  |  |  |
| 2012 | Choking |  |  |  |  |  |
| 2013 | Having his/her teeth cleaned |  |  |  |  |  |
| 2014 | People in white uniforms |  |  |  |  |  |
| 2015 | Having to open his/her mouth wide |  |  |  |  |  |

**SECTION 21**

**MODIFIED CHILD DENTAL ANXIETY SCALE (FACES)**

Ask mothers to estimate the level of fear the child (*for 6-7 years old only*) would perceive in the situations described below. (*If child is 8-12 years, ask the child the questions directly)*

|  | **How do you feel about…** | Not worried | Fairly worried | Very worried |
| --- | --- | --- | --- | --- |
| 2101 | Having your teeth looked at |  |  |  |
| 2102 | Going to the dentist generally |  |  |  |
| 2103 | Having your teeth scraped and polished |  |  |  |
| 2104 | Having a mixture of gas and air which will help you feel comfortable for treatment but cannot put you to sleep |  |  |  |
| 2105 | Having a filling |  |  |  |
| 2106 | Being out to sleep to have treatment |  |  |  |
| 2107 | Having a tooth taken out |  |  |  |
| 2108 | Having an injection in the gum |  |  |  |

**SECTION 22**

**CORAH’S DENTAL ANXIETY SCALE**

Ask mothers to estimate the level of fear the child (*for 6-7 years old only*) would perceive in the situations described below. (*If child is 8-12 years, ask the child the questions directly)*

**Mark your answer with x**

**2201. If your child had to go to the dentist tomorrow, how would (s)he feel about it. Mark the alternative that best describes the feelings**

………. (S)He will look forward to it as a reasonably enjoyable experience

………. (S)He wouldn’t care anyway or the other

………. (S)He would be a little uneasy about it

………. (S)He would be afraid that it would be unpleasant and painful

………. (S)He would be very frightened of what the dentist might do

**2202. When your child is in the waiting room in the dentist’s office for his/her turn in the chair, how would (s)he feel?**

………. Relax

………. A little uneasy

………. Tense

………. Anxious

………. So anxious (s)he may sometimes break out in sweat or almost feel physically sick

**2203. When your child is in the dentist’s chair waiting while he gets his drill ready to begin working on his/her teeth, how does (s)he feel?**

………. Relax

………. A little uneasy

………. Tense

………. Anxious

………. So anxious (s)he may sometimes break out in sweat or almost feel physically sick

**2204. Your child is in the dentist’s chair to have his/her teeth cleaned. While (s)he is waiting and the dentists is getting out the instruments which will be used to scrape his/her teeth around the gun, how does (s)he feel?**

………. Relax

………. A little uneasy

………. Tense

………. Anxious

………. So anxious (s)he may sometimes break out in sweat or almost feel physically sick

**FINAL REMARKS ON THE QUESTONNAIRE**

**INTERVIEW CLOSING TIME………………………….**

**DATE………………………………………………………..**

##### LANGUAGE (s) THAT THE INTERVIEW WAS CONDUCTED …………………………….…………

**INTERVIEWER’S COMMENTS………………………………………………..**

**………………………………………………………………………………………**

**………………………………………………………………………………………**

**………………………………………………………………………………………**

**Name……………………………………………**

**Signature/Date…………………………………………..**

**INFORMATION SHEET FOR STUDY PARTICIPANTS**

**DIGIT SUCKING AND ORAL HEALTH**

If you would like more information, have personal concerns, call  08037234721 or send a mail to kikelomokolawole@gmail.com. The study is being undertaken by the Oral habit study group, Dept of Child Dental Health, OAU/OAUTHC Ile-Ife

**Introduction:** This project is being implemented by a team of researchers from the Department of Child Dental Health, Obafemi Awolowo University/OAUTHC, Ile-Ife. The study is being led by Dr K.A Kolawole. We would like to ask you and your child some questions to find out about certain practices that children engage in and the reasons why. We would also be able to learn from you how best to address these behaviours in this environment.

**Purpose of this study:** This study will try to find out if there is any relationship between oral habits, holes in the teeth and gum problems. It will try to find out what makes children engage in some of these oral habits.

**Procedures:** Persons working on this project will ask you some questions and fill the questionnaire. They will ask you questions about your child’s age, work, feeding and how he/she takes care of his/her mouth. We will also ask you and the father some of these questions. Other questions we will ask include things that you think may make the child fearful, and questions about the health of the child. We would also measure the weight and height of the child, look at the mouth and hand to look for signs of holes in the teeth, gum problems, and signs of having practices any oral habits. All examination in the mouth will be done with gloves and mouth mirrors.

**Benefits:** You will not experience any direct benefit from participation; you may however benefit in the future from information learned from this study.

**Risks**: There are no risks involved in participating in this study. The only discomfort you may have with this study will be the time spent in filling the questionnaire and slight discomfort during intra-oral examination by investigators.

**Compensation:** You will not be paid any compensation for participating in this study.

**Confidentiality:** All study procedures will be conducted in private, and every effort will be made to protect your privacy and confidentiality. Your name will not be written on your questionnaire, and will never be used in connection with any of the information given. Serial numbers will be used. All information we get from you will be stored securely and will only be released to investigators

**Respondents’ Rights:** Your child will only take part in this study if both you and your child agree to participate and sign the consent form. You are also free not to continue with the study if you wish by telling any of the investigators of your decision. There will be no punishment involved if you wish to withdraw from the study.

**Conflict of Interest**: We declare that there are no conflicts of interest.

**CONSENT FORM**

**ASSOCIATION BETWEEN ORAL HABITS AND ORAL HEALTH IN CHILDREN 12 MONTHS TO 12 YEARS RESIDENT IN IFE CENTRAL LOCAL GOVERNMENT AREA.**

***Subject’s Agreement/Consent Form:***

I have read the information provided above, or it has been read to me.

I have had the opportunity to ask questions about it and any questions I have asked have been answered to my satisfaction. I consent voluntarily to participate in this study and understand that I have the right to withdraw from the study at any time.

**Yes No**

**----------------------------------------------------------------------------------------------------**

Signature/Thumb print of Research Respondent. Date:

Printed Name of Research Subject’s Legal Guardian

Signature/thumb print of Person Obtaining Consent. Date:

Printed Name of Person Obtaining Consent.

**ASSENT FORM**

**ASSOCIATION BETWEEN ORAL HABITS AND ORAL HEALTH IN CHILDREN 12 MONTHS TO 12 YEARS RESIDENT IN IFE CENTRAL LOCAL GOVERNMENT AREA.**

***Subject’s Agreement/Assent Form:***

I have read the information provided above, or it has been read to me.

I have had the opportunity to ask questions about it and any questions I have asked have been answered to my satisfaction. I consent voluntarily to permit my Child/Ward to participate in this study and understand that I have the right to withdraw my Child/Ward from the study at any time.

**Yes No**

**----------------------------------------------------------------------------------------------------**

Signature/Thumb print of Research Respondent. Date:

Printed Name of Research Subject’s Legal Guardian

Signature/thumb print of Person Obtaining Consent. Date:

Printed Name of Person Obtaining Consent.

**DATA COLLECTION SHEET**

**Anthropometric measurements**

1. Weight: ………………. kg

2. Height: ……………….. m

**OHI-S (Simplified) - (Greene and Vermillion, 1964)**

**Criteria for calculating Plaque score**

The presence of plaque is verified on the buccal surface of 6 index teeth.

6 1 6 E A E

6 1 6 E A E

0 = no plaque present

1 = plaque covering no more than 1/3 of the surface in question.

2 = plaque covering more than 1/3, but no more than 2/3 of the surface.

3 = plaque covering more than 2/3 of the surface.

**Only fully erupted teeth are scored. There is no substitution for excluded teeth.**

**Criteria for calculating Calculus score**

The presence of calculus is verified on the buccal surface of 6 index teeth.

6 1 6 E A E

6 1 6 E A E

0 = No calculus present

1 = Supragingival calculus extending only slightly below the free gingival margin (not more than 1 mm).

2 = Supragingival calculus covering more than one third but not more than two thirds of the exposed tooth surface OR the presence of individual flecks of subgingival calculus around the cervical portion of the tooth or both.

3 = Supragingival calculus covering more than two thirds of the exposed tooth surface Or a continuous heavy band of subgingival calculus around the cervical portion of the tooth or both.

**Oral hygiene score for child**= Debris+ calculus scores

Number of examined surfaces

0.0 – 1.2 = Good, 1.3 - 3.0 = Fair, > 3.1 = Poor

**PLAQUE INDEX (PI) (Silness and Loe, 1964)**

The six index teeth are

6 2 4 E B D

4 2 6 D B E

Scoring Criteria

0 = No plaque

1 = A film of plaque adhering to the free gingival margin and adjacent area of the tooth, which cannot be seen with the naked eye. But only by using disclosing solution or by using probe.

2 = Moderate accumulations of deposits within the gingival pocket, on the gingival margin and/ or adjacent tooth surface, which can be seen with the naked eye.

3 = Abundance of soft matter within the gingival pocket and/or on the tooth and gingival margin.

**PI SCORE FOR CHILD**:

**GINGIVAL INDEX (GI) (Loe and Silness, 1963)**

The six index teeth are

6 2 4 E B D

4 2 6 D B E

The examination is done by a blunt probe. Partially erupted teeth, retained roots, teeth with periapical lesion and third molars should be excluded and there is no substitution.

**Scoring Criteria**

0 = No inflammation.

1 = Mild inflammation. Slight change in color, slight edema, no bleeding on probing.

2 = Moderate inflammation. Moderate glazing, redness, bleeding on probing.

3 = Severe inflammation. Marked redness and hypertrophy, ulceration, tendency to spontaneous bleeding.

**Gingiva Index score for child** = Total scores

Number of surfaces examined

0.1 -- 1 mild gingivitis; 1.1 -- 2   moderate gingivitis; 2.1 – 3 severe gingivitis

**Dental caries**

1. **Teeth present**

2. **Caries status using dmft/DMFT**:

| 55 | 54 | 53 | 52 | 51 | 61 | 62 | 63 | 64 | 65 |
| --- | --- | --- | --- | --- | --- | --- | --- | --- | --- |
|  |  |  |  |  |  |  |  |  |  |

| 18 | 17 | 16 | 15 | 14 | 13 | 12 | 11 | 21 | 22 | 23 | 24 | 25 | 26 | 27 | 28 |
| --- | --- | --- | --- | --- | --- | --- | --- | --- | --- | --- | --- | --- | --- | --- | --- |
|  |  |  |  |  |  |  |  |  |  |  |  |  |  |  |  |

|  |  |  |  |  |  |  |  |  |  |  |  |  |  |  |  |
| --- | --- | --- | --- | --- | --- | --- | --- | --- | --- | --- | --- | --- | --- | --- | --- |
| 48 | 47 | 46 | 45 | 44 | 43 | 42 | 41 | 31 | 32 | 33 | 34 | 35 | 36 | 37 | 38 |

|  |  |  |  |  |  |  |  |  |  |
| --- | --- | --- | --- | --- | --- | --- | --- | --- | --- |
| 85 | 84 | 83 | 82 | 81 | 71 | 72 | 73 | 74 | 75 |

3. **Caries status using pufa/PUFA**:

| 55 | 54 | 53 | 52 | 51 | 61 | 62 | 63 | 64 | 65 |
| --- | --- | --- | --- | --- | --- | --- | --- | --- | --- |
|  |  |  |  |  |  |  |  |  |  |

| 18 | 17 | 16 | 15 | 14 | 13 | 12 | 11 | 21 | 22 | 23 | 24 | 25 | 26 | 27 | 28 |
| --- | --- | --- | --- | --- | --- | --- | --- | --- | --- | --- | --- | --- | --- | --- | --- |
|  |  |  |  |  |  |  |  |  |  |  |  |  |  |  |  |

|  |  |  |  |  |  |  |  |  |  |  |  |  |  |  |  |
| --- | --- | --- | --- | --- | --- | --- | --- | --- | --- | --- | --- | --- | --- | --- | --- |
| 48 | 47 | 46 | 45 | 44 | 43 | 42 | 41 | 31 | 32 | 33 | 34 | 35 | 36 | 37 | 38 |

|  |  |  |  |  |  |  |  |  |  |
| --- | --- | --- | --- | --- | --- | --- | --- | --- | --- |
| 85 | 84 | 83 | 82 | 81 | 71 | 72 | 73 | 74 | 75 |

**O’sullivan index for measurement of dental erosion:**

The central incisors, lateral incisors, and first molars in the upper and lower jaws will be examined. The index of O’Sullivan (O’Sullivan *et al* 1998) will be adopted to record the distribution, severity, and amount of affected teeth.

6 2 1 1 2 6 E B A A B E

6 2 1 1 2 6 E B A A B E

**Site on erosion on each tooth**

Code A: Labial or buccal only

Code B: Lingual or palatal only

Code C: Occlusal or incisal only

Code D: Labial and incisal/occlusal

Code E: Lingual and incisal/occlusal

Code F: Multi-surface

**Grade of severity (worst score for an individual tooth recorded)**

Code 0: Normal enamel

Code 1: Matt appearance of the enamel surface with no loss of contour

Code 2: Loss of enamel only (loss of surface contour)

Code 3: Loss of enamel with exposure of dentine (enamel-dentin junction visible)

Code 4: Loss of enamel and dentine beyond enamel dentine junction

Code 5: Loss of enamel and dentine with exposure of the pulp

Code 9: Unable to assess (e.g. tooth crowned or large restoration)

**Area of surface affected by erosion**

Code -: Less than half of surface affected

Code +: More than half of surface affected

ORTHODONTIC EXAMINATION

| Teeth present |  |  |  |  |
| --- | --- | --- | --- | --- |
| 1. Facial profile |  | Straight | Convex | Concave |
| 2. Lips |  | Competent | Potentially competent | Incompetent |
| 3. Jackson lip classification |  |  |  |  |
| 4. Skeletal relationship |  | Class 1 | Class 2 | Class 3 |
| 5. Overjet | ___ (mm) | Normal | Increased | Increased |
| 6. Overbite | ____ (mm) | Normal | Deep bite | Incomplete bite |
| 7. Anterior open bite | ___ (mm) | Unilateral | Bilateral |  |
| 8. Posterior open bite | ___ (mm) | Unilateral | Bilateral |  |
| 9. Canine relationship |  | Class I | Class I1 | Class I11 |
| 10. Primary second molar relationship |  | Flush | Mesial | Distal |
| 11. First Molar relationship |  | Class 1 | Class II | Class III |
| 12. Anterior cross bite | ____ (mm) | Absent | Unilateral | Bilateral |
| 13. Buccal cross bite |  | Absent | Unilateral | Bilateral |
| 14. Lingual cross bite |  | Absent | Unilateral | Bilateral |
| 15. Upper anterior inclination |  | Normal | Proclined | Retroclined |
| 16. Lower anterior inclination |  | Normal | Proclined | Retroclined |
| 17. Upper anterior crowding | --- (mm) | Mild | Moderate | Severe |
| 18. Upper anterior spacing | ------ (mm) | Absent | Present |  |
| 19. lower anterior spacing | ------ (mm) | Absent | Present |  |

Examination of upper extremities in the case of finger habits to check for presence or absence of

|  |  |  |
| --- | --- | --- |
| Clean digit | Yes | No |
| Redness, wrinkling chapped or blistered finger | Yes | No |
| Dishpan thumb | Yes | No |
| Fibrous roughened wart like callus on superior aspect of digit | Yes | No |
| Ulceration | Yes | No |
| Corn formation | Yes | No |
| Finger deformity | Yes | No |
| Damage to nail and nail bed | Yes | No |
